# Terpenoids: A Comprehensive Review on Biosynthesis, Applications and Enhancement Strategies for Translational Research

**DOI:** 10.3390/ijms27209280

**Published:** 2026-10-19

**Authors:** Irum, Gyanendra Tripathi, Suhail Ahmad, Nida Fatima, Paridhi Puri, Naved Ahmad, Kasun Kumara Dissanayake, Khurshid Ahmad, Saheem Ahmad, Alvina Farooqui

**Affiliations:** 1 Department of Biosciences, Integral University, Lucknow 226026, India or irumphd@student.iul.ac.in; 2 Department of Bioengineering, Integral University, Lucknow 226026, India; 3 University Center for Research and Development, Chandigarh University, Mohali 140413, India; 4 College of Medical Sciences, AlMaarefa University, Diriyah, Riyadh 13713, Saudi Arabia; 5 Department of Chemical Engineering and Biotechnology, Karshi State Technical University, Karshi 180100, Uzbekistan; kasunkumar86@gmail.com; 6 Department of Health Informatics, College of Applied Medical Sciences, Qassim University, Buraydah 51452, Saudi Arabia; 7 Department of Medical Laboratory Sciences, College of Applied Medical Sciences, University of Hail, Hail City P.O. Box 2440, Saudi Arabia; ahmadsaheem@gmail.com

**Keywords:** isoprene, terpenoids, CRISPR-Cas system, clinical trials, elicitors, nanotechnology, bioavailability, metabolic engineering

## Abstract

There is growing interest in natural bioactive compounds as potentially safer and more sustainable sources of therapeutic agents. Terpenoids, one of the most structurally diverse and biologically active classes of natural products, are derived from repeating isoprene units and possess a broad range of pharmacological and industrial applications. This review provides a comprehensive overview of recent advances in terpenoid biosynthesis, structural classification, and therapeutic applications. It also discusses emerging approaches for enhancing large-scale production, improving functional efficacy, and overcoming pharmacokinetic limitations. Attention is given to terpenoids currently undergoing clinical evaluation, along with their progress toward commercial development. Despite substantial preclinical evidence supporting their therapeutic potential, the clinical translation of many terpenoids remains limited. Key challenges include poor aqueous solubility, low permeability, slow dissolution, and high lipophilicity, which can collectively restrict systemic bioavailability and therapeutic efficacy. Moreover, only a relatively small proportion of biologically active terpenoids have progressed to advanced stages of clinical development, highlighting a considerable gap between experimental findings and clinical application. This review integrates current knowledge of terpenoid biosynthesis, biological activities, production strategies, and translational development while highlighting sustainable and scientifically driven approaches that may facilitate their future therapeutic and commercial utilization.

## 1. Introduction

Terpenes, a structurally diverse class of natural compounds, are biosynthesized from isoprene units, which serve as the fundamental building blocks. These compounds are ubiquitously present in nature predominantly as secondary metabolites in plants (e.g., essential oils, resins, waxes and latex) and are also synthesized by various bacteria, fungi and algae [1]. The polymerization of isoprene units through head-to-tail linkages leads to the formation of linear or cyclic structures that constitute the backbone of terpenoid diversity [2].

Terpenoids are derived from basic terpene structures through functional group modifications, such as oxidation and molecular rearrangement, and are commonly classified according to the number of isoprene units they contain. Major classes include monoterpenes (C10), sesquiterpenes (C15), diterpenes (C20), triterpenes (C30), and tetraterpenes (C40) [2]. A wide range of pharmacological activities has been attributed to terpenoids, including anticancer, antioxidant, anti-inflammatory, and antidiabetic effects [3]. For instance, terpenoids such as oleanolic acid and lupeol have been reported to improve insulin sensitivity and reduce blood glucose levels, while several sesquiterpenes exhibit potent anti-inflammatory activity, which may be beneficial in the management of diabetes and its associated complications [4].

Beyond terrestrial ecosystems, terpenoid emissions are also associated with marine environments, where phytoplankton blooms and marine algae represent important biological sources of monoterpenes and contribute to the release of biogenic volatile organic compounds into the atmosphere. Interestingly, larger terpenes containing multiple isoprene units may exhibit greater thermal stability than certain other secondary metabolites, which could support their use in a range of environmental and industrial applications [5].

Historically valued for their contribution to the fragrance of flowers and the flavor of fruits, terpenoids have attracted considerable attention from pharmacologists, ecologists, and industry because of their structural diversity, broad biological activities, and potential for sustainable applications. In recent years, interest in these compounds has expanded considerably, particularly in relation to their therapeutic potential and applications in agriculture, nutraceuticals, and sustainable industrial processes. This growing interest is supported by increasing scientific evidence demonstrating their potential roles in drug discovery, disease management, and the development of environmentally compatible technologies.

The biosynthetic routes responsible for terpenoid production include two major pathways: the mevalonic acid (MVA) pathway and the 2-C-methyl-D-erythritol-4-phosphate (MEP) pathway [6]. Based on their metabolic roles, terpenoids are further classified as primary metabolites, such as quinones, photosynthetic pigments, sterols, growth hormones and prenylated proteins, or as secondary metabolites, which are involved in ecological defense and communication [7]. One vital role is protein prenylation, a post-translational modification that facilitates membrane localization and function of signaling proteins, such as GTPase [8]. Crucial intermediates like farnesyl diphosphate (FPP) and geranyl diphosphate (GPP), generated during terpenoid biosynthesis, perform essential biological functions, including pollinator attractions, natural pest resistance, antibacterial activity and allelopathic interactions, while also exhibiting antioxidant properties [7].

Despite the enormous biological potential of terpenoids, their commercial exploration remains limited due to low natural abundance, the slow growth of source organisms, environmental vulnerability and variability in compound concentrations [9]. Several valuable terpenoids are produced in trace amounts while others come from endangered species, precluding large-scale extraction [10]. Moreover, the chemical synthesis of complex terpenoids is often cost-prohibitive and labor-intensive. These limitations have prompted a surge in the development of production enhancement strategies, such as elicitor additions, metabolic engineering, organelle engineering and advanced genome editing technologies, like CRISPER-Cas systems. These approaches have significantly improved the yield and scalability of terpenoid production, thereby addressing the bottleneck between research and industrial applications. Although terpenoids exhibit a broad spectrum of biological activities, their practical use in pharmaceutical and industrial settings remains limited. Challenges such as low extraction yields, poor solubility and inadequate systemic absorption continue to restrict their effectiveness. Moreover, only a small number of these compounds have progressed to clinical evaluations, reflecting a noticeable gap between laboratory research and therapeutic applications.

Furthermore, the gaps between laboratory-scale research and the industrial or clinical translation of terpenoids have not been adequately addressed. Bridging these gaps is essential for the rational advancement of terpenoids from experimental compounds to viable therapeutic and industrial products. In this context, the present review provides a comprehensive and integrative overview of the biosynthesis of terpenoids, their diverse applications, and emerging strategies to overcome the major challenges associated with their production and utilization, with particular emphasis on translational relevance. By critically examining recent advances, existing limitations, and future opportunities, this review seeks to bridge the gap between fundamental research and the practical application of terpenoids in the pharmaceutical, agricultural, and sustainable industrial sectors.

## 2. Literature Search Strategy

A comprehensive literature search was conducted to identify relevant publications on terpenoids, encompassing their classification, biosynthesis, medicinal applications, agricultural and industrial uses, strategies for enhancing production yields, and approaches to improving bioavailability. The relevant literature was retrieved from electronic databases, including PubMed and Scopus. The search primarily covered studies published between January 2010 and September 2026.

An initial PubMed search using the keyword “Terpenoids” yielded 5498 records. These publications were subjected to a multistage screening process to identify studies relevant to the objectives of the present review. Following the initial title screening, 3540 articles were retained for further evaluation. Subsequent abstract screening reduced this number to 2165 articles. Full-text assessment resulted in the identification of 536 potentially relevant publications. Following a final evaluation of their scientific relevance, methodological rigor, and contribution to the scope of the review, 240 articles were selected for inclusion in the final narrative synthesis.

To ensure comprehensive coverage of the major themes addressed in this review, additional targeted searches were performed using combinations of keywords aligned with the specific review objectives. The PubMed search term “Terpenoid classification and biosynthesis” retrieved 50 articles within the specified publication period, whereas “Terpenoids AND Pharmacological applications” yielded 146 records. Searches using “Terpenoids AND Industrial applications” and “Terpenoids AND Agricultural applications” each identified 37 articles. Furthermore, the search term “Terpenoids AND Enhancement strategies” retrieved 56 publications. These targeted searches were used to identify additional relevant evidence and support the thematic organization of the review.

The selected literature was critically evaluated and synthesized to provide an integrated overview of advances in terpenoid biosynthesis, biological activities, industrial and agricultural applications, production enhancement strategies, and translational challenges. Particular emphasis was placed on recent developments, emerging technologies, and research gaps that may inform future investigations and facilitate the translation of terpenoid-based discoveries into practical applications.

Initially, the keywords “terpenoid” AND/OR “isoprenoid” were utilized in the search. Then, other keywords and search terms were used depending on the theme under which the review was to be conducted. Some of the themes that were used were “Terpenoid classification”, “Terpenoid biosynthesis”, “Terpenoid AND pharmaceutical applications”, “Terpenoid AND agricultural applications”, “Terpenoid AND industrial applications”, “Terpenoid production enhancement”, and “Terpenoid bioavailability”. Relevant full-text publications were evaluated for their scientific significance, relevance to the respective sections and contribution to the understanding of terpenoid research. Additional relevant studies were identified through targeted keyword searches and examination of the reference lists of selected publications. The literature selection was guided by thematic relevance and scientific contribution. The resulting literature was synthesized to provide an integrated overview of terpenoids, from biosynthesis and applications to production enhancement and bioavailability strategies.

## 3. Terpenes and Terpenoids

Terpenes are naturally occurring hydrocarbons composed of repeating isoprene units, each containing five carbon atoms. These compounds form the structural basis of essential oils and are characterized by carbon skeletons derived from 2-methylbuta-1,3-diene (isoprene) units, which can undergo rearrangements to generate a variety of cyclic structures. Examples include myrtenol, verbenone, p-cymen-8-ol, γ-terpinen-7 al, carvone, and β-thujone, all of which are present in the essential oils of various plant species [11].

Terpenoids, also known as isoprenoids, are chemically modified derivatives of terpenes that undergo processes such as oxidation, molecular rearrangement or functional group substitution. These modifications result in diverse chemical forms, including ketones, alcohols, acids, ethers, esters, and aldehydes. With more than 80,000 known compounds, terpenoids represent the largest class of secondary metabolites and contribute significantly to plant metabolism [12]. They are commonly found in essential oils and classified into several categories, such as cardiac glycosides, saponins, true terpenes, and steroids [13]. Many terpenoids exhibit valuable pharmacological properties, which enhance their importance in medicinal chemistry [14]. Notable examples include menthol, thymol, linalool, geraniol, citronellal, carvacrol, and piperitone.

The chemical structure basis of terpenes and terpenoids lies in the isoprene rule, which was first recognized by Greville Williams in 1859 and later refined by Wallach and Ruzicka in the 20th century [15]. This principal concept suggests that terpenes are constructed from isoprene units linked in either a regular or irregular fashion [16]. Isoprenoids perform critical biological functions, including contributing to the side chains of archaeal lipid membranes and supporting photosynthesis through roles in light absorption, electron transport, and energy conversion in plants [14]. Terpenoids hold substantial industrial importance, with wide-ranging applications in the pharmaceutical, fragrance and flavor industries and in agrochemical formulations [17]. Moreover, monoterpenes, owing to their highly volatile nature at elevated temperatures, play a key role in determining the composition and concentration of volatile compounds in plant tissues [18]. Terpenoids are widely distributed across animals, plants and microalgae, where they serve critical ecological, physiological and defensive functions [19]. Plants represent the most prolific natural reservoir producing clinically and industrially valuable terpenoids, such as artemisinin and paclitaxel, alongside carotenoids [20]. Microalgae also contribute as a promising source of terpenoid production due to their high photosynthetic efficiency, metabolic flexibility and capacity to convert CO_2_ into high-value terpenoids such as β-carotene, astaxanthin, fucoxanthin and lutein. Their scalability, sustainability and rich chemical diversity position microalgae as an attractive platform for future terpenoid-based biotechnological and industrial applications.

The extraction process is an essential stage in the isolation of terpenoids from medicinal plants and marine algae, as the extraction parameters can impact not only the yield of terpenoids but also their chemical composition. Traditional methods such as maceration, Soxhlet extraction, solvent extraction, hydrodistillation, and steam distillation are still commonly used due to their simplicity and convenience. However, traditional methods may take a long time and require high amounts of solvents and heat exposure, which negatively impact the yield of volatile or thermolabile terpenoids [21]. Novel extraction techniques, such as ultrasound-assisted extraction (UAE), microwave-assisted extraction (MAE), supercritical fluid extraction (SFE), pressurized liquid extraction (PLE), and enzyme-assisted extraction (EAE), are now more effective. In particular, UAE accelerates mass transfer by the cavitation effect and allows for decreasing the extraction time and solvent usage, while MAE involves the fast heating of samples and the release of intracellular compounds. SFE is appropriate for non-polar terpenoids and volatile fractions, while PLE allows for extracting compounds under controlled temperature and pressure conditions [22]. In the case of marine algae, extraction efficacy depends on the complexity of the cellular matrix, moisture, and the co-extraction of lipids and pigments. UAE, MAE, SFE, and subcritical water extraction techniques have attracted growing interest among researchers as a way to increase the efficacy of extracting bioactive substances from marine algae. The optimization of factors such as the solvent polarity, temperature, time of extraction, pressure, solid-to-solvent ratio, and pretreatment is necessary, as they affect not only the extraction efficacy but also the amounts of certain metabolites [23].

## 4. Classification of Terpenoids

A considerable portion of secondary metabolites belongs to the terpenoid family, which is known for its extensive structural diversity. Terpenes are classified into subgroups based on the number of carbon atoms they contain, which, in turn, depends on the number of isoprene units present. As the number of units increases, so does the complexity and classification of terpenes [24]. Table 1 represents the sub-classes of terpenoids with examples.

### 4.1. Hemiterpenoids

Hemiterpenes constitute a relatively small subset of the terpene components found in essential oils. They are typically built upon a 2-methylbutane skeleton and are often functionalized with alcoholic, aldehyde, and ester functional groups [25]. Compared to other terpene classes, hemiterpenes are less prevalent in essential oils, with fewer than 100 known compounds reported to date [26]. Notably, the leaves of *Prinsepia utilis* have been identified as a source of chlorinated hemiterpenes [27]. Recent phytochemical studies led to the isolation of new hemiterpenoid derivatives, namely, Taxilluside A to D (designated as 1 to 4), from the branches and foliage of *T*. *chinensis* [28]. Their biological potential was evaluated based on their ability to modulate intracellular calcium levels in myocardial cells, assessed via changes in fluorescence intensity using a Laser Scanning Confocal Microscope (LSCM) [28]. In addition to their biological significance, hemiterpenes, especially isoprene, play an important role in atmospheric chemistry. Isoprene emissions, primarily biogenic in origin, are highly reactive with atmospheric gases [29]. When exposed to anthropogenic nitrogen oxides and sunlight, isoprene undergoes photochemical degradation, leading to the formation of tropospheric ozone. Furthermore, isoprene reacts directly with ozone to generate products such as methacrolein, formaldehyde, and methyl vinyl ketone. This reaction also yields moderate levels of H_2_O and other reactive oxidants [30].

**Table 1:** Classification of terpenoids.

No. of Carbon Atoms	No. of Isoprene Units	Class	Plant Species	Examples	Reference
C5	1	Hemiterpenes	*Arabidopsis thaliana*, *Populus* spp.	Isoprene	[31]
C10	2	Monoterpenes	*Mentha piperita*, *Pinus* spp., *Lavandula angustifolia*	Menthol, limonene, α-pinene	[32]
C15	3	Sesquiterpenes	*Gossypium hirsutum*, *Artemisia annua*	Gossypol, artemisinin	[33]
C20	4	Diterpenes	*Taxus brevifolia*, *Gibberella fujikuroi*	Taxol, gibberellic acid	[34]
C30	6	Triterpenes	*Arabidopsis thaliana*, *Avena sativa*, *Glycyrrhiza glabra*	Sitosterol, oleanolic acid	[35]
C40	8	Tetraterpenes	*Daucus carota*, *Solanum lycopersicum*, *Dunaliella salina*	β-carotene, lycopene	[36]
>C40	>8	Polyterpenes	*Hevea brasiliensis*, *Arabidopsis thaliana*	Natural rubber, plastoquinone	[37]

### 4.2. Monoterpenoids

Monoterpenes are a class of terpenoids consisting of ten carbon atoms (C_10_H_16_), typically formed by the union of two isoprene units. The compounds often exhibit aromatic properties and play a vital role in the pharmaceutical industry due to their bioactive potential. They are highly volatile and have thus become key constituents of essential oils extracted from various plant species [38]. Based on their structure, monoterpenes are classified into two major groups: acyclic monoterpenes, which feature an open carbon chain structure (such as myrcene and ocimene), and cyclic monoterpenes, which can contain one or more rings (like limonene) or multiple cycles (including fenchanes and pinenes) (Table 1). Additionally, some bicyclic monoterpenes incorporate cyclopropane rings (for instance, sabinene). These compounds form the principal components of essential oils derived from fruits and contribute to the volatile turpentine fraction in the oleoresins of coniferous trees. Aromatic aldehydes, like geranial, emit a citrus aroma and are found in lemon essential oils. Camphor, a well-known bicyclic monoterpene, is a major compound found in the essential oils of rosemary, lavender, and sage [39].

### 4.3. Sesquiterpenoids

Sesquiterpenoids represent a crucial class of terpenes composed of three isoprene units, in the molecular formula C_15_H_24_. These compounds exhibit a wide range of structural forms, including linear chains, monocyclic and polycyclic structures, and lactone rings. In many plant species, sesquiterpenes are abundant in latex and are known for their pronounced antimicrobial and insecticidal activities [40]. One notable example is Artemisinin (Table 1), a highly potent sesquiterpene lactone extracted from the aerial parts and roots of *Artemisia annua* [25]. Sesquiterpenoids are predominantly found in higher plants but are also reported in a variety of other biological sources, including marine fungi and other marine organisms [41]. They occur either as hydrocarbons or in oxygenated form, such as ketones lactones, acids, alcohols, and aldehydes. With over 5000 known compounds, they form a substantial portion of plant secondary metabolites [42].

### 4.4. Diterpenoids

Diterpenes are a class of terpenes composed of four isoprene units, in the molecular formula C_20_H_32_. These compounds often contain multiple O_2_ atoms in the form of ketonic or hydroxyl groups and can exhibit either linear or cyclic structural configurations. Biologically significant diterpenes include retinol, retinal, and phytol, which are known for their essential roles in vision, cellular signaling, and photosynthesis [43]. Several diterpenes, such as grayanotoxin, eleganolone, and marrubenol, are recognized for their notable effects on the cardiovascular system [44]. Structurally, diterpenes may be organized into a variety of rings, including linear, bi-, tri-, tetra-, penta-, and macrocyclic arrangements, depending on the complexity of their carbon backbone. Interestingly, in marine ecosystems, certain invertebrates, such as sponges, polyps, seaweeds, and bacteria, form symbiotic or mutualistic associations involving diterpene compounds. These marine organisms often produce or harbor diterpene alcohol, which serves as natural camouflage or a chemical defense mechanism (a phenomenon sometimes referred to as ‘clothing’). This biochemical adaptation helps protect invertebrates from predation by fish and other marine predators [45].

### 4.5. Triterpenoids

Triterpenes are a group of secondary metabolites characterized by a 30-carbon skeleton constructed from six isoprene units and represented by the molecular formula (C_30_H_48_) (Table 1). These compounds often contain oxidizable methyl (CH_3_) groups within their structure. Among them, saponin glycosylated triterpenes are well documented for their wide-ranging biological activities [43]. Biosynthetically, triterpenes are derived from squalene, a C30 building block composed of six C5 isoprene units. Through enzymatic cyclization, squalene gives rise to a diverse array of triterpenoids, including saponins (glycosides), steroids, and sterols [46]. The triterpenoid class comprises more than 20,000 known compounds, reflecting significant structural diversity and functional versatility [47]. Triterpenoids play essential physiological roles at both the cellular and organ systematic levels. They are integral components of cellular membranes and serve as biochemical precursors for critical signaling molecules, such as steroid hormones and their corresponding receptors. For example, after cyclization, squalene produces lanosterol, a precursor to cholesterol and ergosterol, which is involved in the biosynthesis of β-sitosterol [46]. In addition to their structural roles, triterpenes and their derivatives demonstrate a wide range of biological activities. These include potential therapeutic effects against cancer neurodegenerative disease, aging-related conditions, inflammatory disorders, metabolic syndromes, and cardiovascular disease, as well as antimicrobial properties against fungal, bacterial, and viral pathogens [47].

### 4.6. Tetraterpenoids

Carotenoids are naturally occurring pigments that are classified as tetraterpenoids. They possess a distinctive C40 hydrocarbon backbone in their molecular structure [48]. These compounds are primarily divided into two categories based on the presence or absence of oxygen atoms: (i) xanthophylls, which are oxygenated derivatives, such as astaxanthin, fucoxanthin, and lutein, and (ii) carotenoids, which lack oxygen and include α-carotene, β-carotene, and lycopene. Additionally, carotenoids are classified into two functional groups depending on their ability to serve as provitamin A sources. They overpower photosynthetic stress through the neutralization of reactive oxygen species (ROS) formed under intense light conditions. Approximately 600 unique carotenoid compounds have been identified, of which nearly 40 are commonly ingested through the human diet. In European nations, lutein and β-carotene are among the most frequently consumed dietary carotenoids. Lutein is abundantly found in various green leafy vegetables, such as parsley, zucchini, asparagus, broccoli, lettuce, kale, and spinach. β-carotene, which acts as a provitamin A compound, is one of the most prevalent carotenoids in nature and is present in a wide range of fruits and vegetables, including apricot, spinach, lettuce, pepper, and kale [49].

## 5. Biogenetic Routes of Terpenoid Synthesis

**Figure 1:**
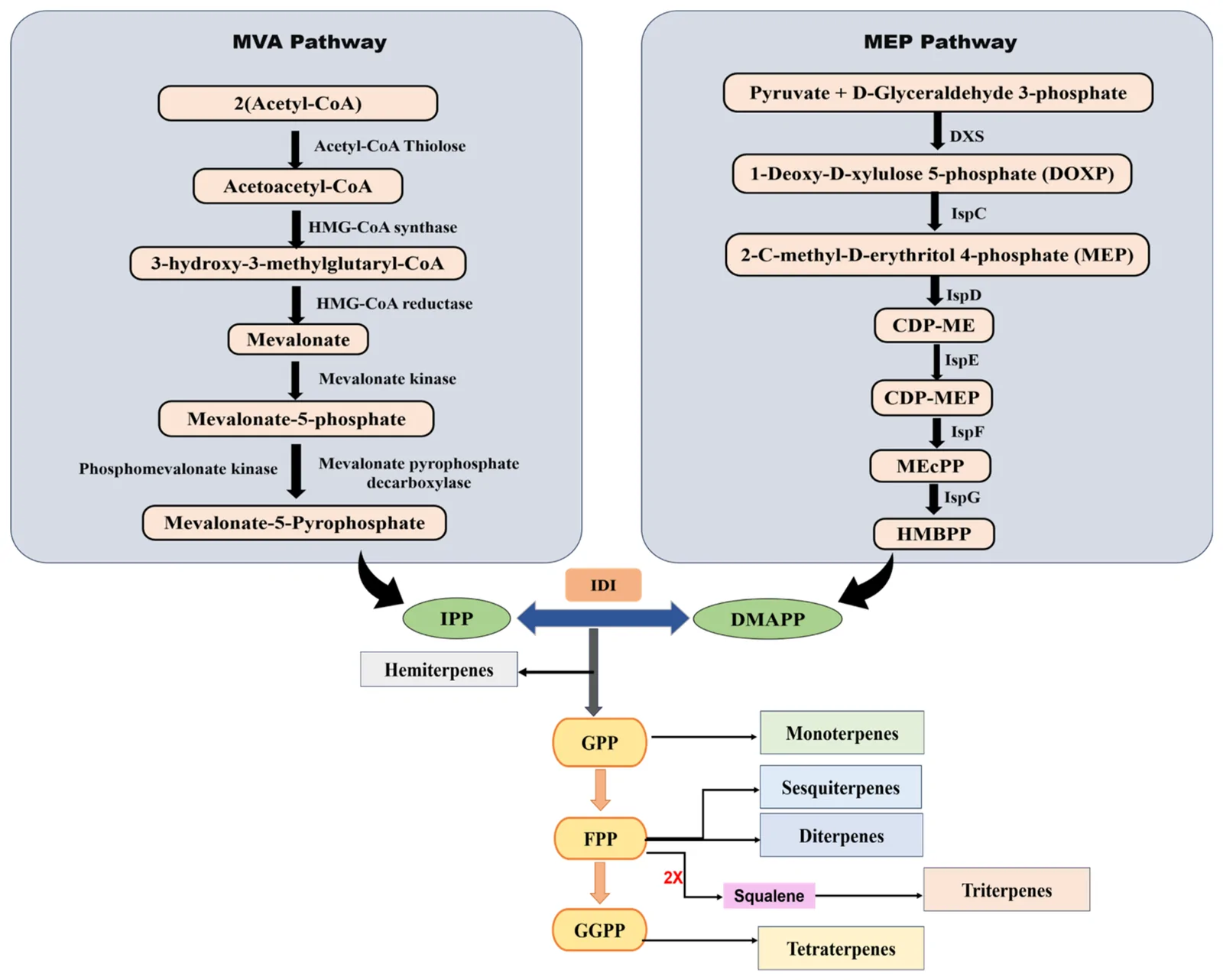
Terpenoid biosynthesis pathways: MEP and MVA pathways.

Terpenoids represent one the most abundant and functionally significant classes of bioactive compounds, widely recognized for their roles in addressing various biological and industrial issues. Given their importance, understanding the mechanism underlying their biosynthesis is essential. Currently, two primary metabolic routes have been identified for terpenoid synthesis: (a) the MEP (2-C-methyl-erythritol 4-phosphate) pathway, also known as the DXP (1-deoxy-d-xylulose 5-phosphate) pathway or the non-mevalonate pathway, and (b) the mevalonate pathway (MVA) (Figure 1). These distinct biosynthesis routes activate in different cellular compartments and are critical for the production of a wide range of terpenoid molecules. Bacteria, particularly Gram-negative strains along with cyanobacteria and some other algae, predominantly utilize the MEP pathway; in contrast, archaeal and eukaryotic organisms, such as Gram-positive bacteria and yeasts, employ the MVA pathway [50]. Interestingly both biosynthetic routes are present in plants and animals. However, in plants and algae, these pathways operate with the MEP pathway localized in plastids and the MVA pathway operating within the cytosol. These plastid-localized pathways are believed to have been inherited from ancestral cyanobacterial endosymbionts. Both the MEP and MVA pathways lead the formation of two essential precursors— isopentenyl pyrophosphate (IPP) and dimethylallyl pyrophosphate (DMAPP)—which are central to the synthesis of all terpenoids. While these end products are the same, the pathways differ significantly in their initial substrates: acetyl-CoA serves as the starting compound in the MVA pathway, whereas the MEP pathway begins with glyceraldehyde-3-phosphate and pyruvate [51]. In terms of efficiency, the MEP pathway converts approximately 83% of the carbon substrate into IPP, whereas the MVA pathway achieves a lower yield of about 56% from an energy standpoint. The MVA pathway is comparatively more productive, generating one molecule each of ATP and either NADPH or NADH_2_ per glyceraldehyde-3-phosphate molecule. The MEP pathway yields only a single molecule of ATP [52]. These two pathways also direct the synthesis of different classes of terpenoids: the MVA pathway primarily contributes to the formation of sesquiterpenes and polyterpenes, while the MEP pathway supports the synthesis of monoterpenes, diterpenes, and carotenoids [53]. Terpenoids exhibit incredible diversity, which is a result of the biochemical transformation of simple prenyl diphosphate molecules. Once the diphosphate group undergoes ionization, an allylic carbocation initiates a cascade reaction, leading to the formation of hydrocarbon backbones (both linear and cyclic). Subsequent modifications with functional groups and substituent groups make terpenoids suitable for use in various industries [54].

A study investigating the biosynthetic pathways in *Salvia miltiorrhiza* hairy roots evaluated the relative contributions of the MVA and MEP pathways to root growth and tanshinone production. To investigate the involvement of these pathways, specific metabolic inhibitors were employed. The results showed that mevinolin, an inhibitor of the MVA pathway, suppressed hairy root growth more strongly than fosmidomycin, an inhibitor of the MEP pathway. Although inhibition of either pathway resulted in a significant reduction in tanshinone accumulation, fosmidomycin exerted a greater inhibitory effect on tanshinone production than mevinolin. Furthermore, treatment with D,L-glyceraldehyde, which interferes with the intracellular transport of isopentenyl pyrophosphate (IPP), enhanced the inhibitory effects of both mevinolin and fosmidomycin on tanshinone biosynthesis and hairy root growth [55]. These findings suggest that both the MVA and MEP pathways contribute to tanshinone biosynthesis in *S. miltiorrhiza*, with possible metabolic interactions and exchange of isoprenoid precursors between the two pathways.

The MEP pathway, also known as the non-mevalonate or methylerythritol phosphate pathway, is an important metabolic pathway found in a wide range of organisms, including in many bacteria and plastid-containing organisms, such as plants and algae (Figure 1). The pathway is responsible for the biosynthesis of two key isoprenoid precursors, isopentenyl pyrophosphate (IPP) and dimethylallyl pyrophosphate (DMAPP), which serve as essential building blocks for the synthesis of terpenoids and several other biologically important compounds.

The MEP pathway begins with the condensation of glyceraldehyde-3-phosphate (G3P) and pyruvate to form 1-deoxy-D-xylulose-5-phosphate (DXP), a reaction catalyzed by DXP synthase (DXS). DXP is subsequently converted to 2-C-methyl-D-erythritol-4-phosphate (MEP) by DXP reductoisomerase (DXR) through an NADPH-dependent reduction and rearrangement reaction. MEP is then converted to 4-diphosphocytidyl-2-C-methyl-D-erythritol (CDP-ME) by CDP-ME synthase (CMS/IspD). CDP-ME is subsequently phosphorylated by CDP-ME kinase (CMK/IspE) to form 4-diphosphocytidyl-2-C-methyl-D-erythritol-2-phosphate (CDP-ME2P).

In the subsequent steps, CDP-ME2P is converted by ME-cPP synthase (MCS/IspF) into 2-C-methyl-D-erythritol-2,4-cyclodiphosphate (ME-cPP). This intermediate is further converted by HMBPP synthase (HDS/IspG) into 4-hydroxy-3-methylbut-2-enyl diphosphate (HMBPP). Finally, HMBPP reductase (HDR/IspH) catalyzes the formation of IPP and DMAPP. These two intermediates subsequently enter multiple biosynthetic routes, leading to the formation of a diverse range of isoprenoid compounds.

The MEP pathway therefore plays a central role in cellular metabolism by supplying the precursors required for the biosynthesis of terpenoids and, in photosynthetic organisms, important compounds such as chlorophyll side chains and carotenoids [56].

The mevalonate (MVA) pathway is a major metabolic pathway predominantly found in animals, fungi, and some archaea, where it plays an essential role in the biosynthesis of isoprenoids. The pathway begins with the sequential condensation of acetyl-CoA molecules. Initially, two molecules of acetyl-CoA condense to form acetoacetyl-CoA, which subsequently reacts with a third acetyl-CoA molecule to produce 3-hydroxy-3-methylglutaryl-CoA (HMG-CoA). HMG-CoA is then reduced to mevalonate by HMG-CoA reductase (HMGCR) in an NADPH-dependent reaction. HMG-CoA reductase serves as a major regulatory enzyme and catalyzes the rate-limiting step of the pathway, making it particularly important in the regulation of cholesterol biosynthesis.

Mevalonate subsequently undergoes a series of ATP-dependent phosphorylation reactions, followed by decarboxylation, to generate isopentenyl pyrophosphate (IPP), a fundamental five-carbon precursor of isoprenoid biosynthesis. IPP can be isomerized to dimethylallyl pyrophosphate (DMAPP), and these two intermediates serve as building blocks for the synthesis of larger isoprenoid molecules through successive condensation reactions. The MVA pathway therefore provides essential precursors for the biosynthesis of numerous biologically important compounds, including cholesterol, steroid hormones, bile acids, and vitamin D, as well as other isoprenoid-derived cellular components.

The MVA pathway is tightly regulated at the level of HMG-CoA reductase, which is subject to feedback inhibition and modulation by hormonal signals. The regulatory control is of particular clinical relevance, as HMG-CoA reductase is the molecular target of statins, widely prescribed medications used to lower elevated cholesterol levels in patients with hypercholesterolemia. Given its essential role in the synthesis of critical biomolecules, the MVA pathway remains a significant focus of biochemical and pharmaceutical research [50].

## 6. Utilizations of Terpenoids in Various Sectors

The terpenoid class possesses a wide range of chemical and biological properties. This versatile applicability has made these compounds favorable for various sectors of research, including pharmaceutical, agriculture, food science and environmental studies. Below are listed some sectors utilizing terpenoids for the betterment of mankind.

### 6.1. Pharmaceutical Application of Terpenoids

Terpenoids are powerful compounds that have been used against disease and disorders from ancient times to the present, irrespective of their source of origin. There are many types of terpenoids with various types of efficacies, like cardioprotective, antiviral, antioxidant, and anti-inflammatory efficacies. Some of the pharmaceutical applications are listed below.

#### 6.1.1. Terpenoids as Traditional Medicines

Terpenoids have played a fundamental role in traditional medicine practices across diverse cultures throughout history. Indigenous cultures and traditional healers have long utilized therapeutic, rich botanical properties to treat a wide range of ailments, and these ethnobotanical applications have significantly informed modern pharmaceutical discovery efforts [57]. One notable example is the Artemisia genus, particularly *Artemisia annua*, which has been employed in traditional Chinese medicine for over two millennia to treat febrile conditions [58]. The isolation and structural characterization of the sesquiterpene lactone artemisinin from Artemisia annua marked a breakthrough in antimalarial therapy, culminating in the awarding of the 2015 Nobel Prize in Medicine to Youyou Tu [59]. Furthermore, essential oils derived from terpenoid-rich plants such as *Mentha piperita* (peppermint), *Eucalyptus* spp. (eucalyptus), and *Melaleuca alternifolia* (tea tree) have been widely used in traditional systems for their antimicrobial, anti-inflammatory, and analgesic properties [60]. The leaves of *Diospyros kaki*, a plant rooted in traditional Chinese medicine, contain three bioactive triterpenes—barbinervic acid, its epimer rotungenic acid, and 24-hydroxy ursolic acid—each known for their therapeutic potential. Moreover, limonene, a monoterpenoid found in Yuzu (a citrus fruit used in Japanese medicine), has demonstrated promising antimicrobial, anti-inflammatory, and analgesic properties for the management of bronchial asthma [61]. Also, wild plants such as *Origanum vulgare* possess terpenoids and alkaloids and have demonstrated better antioxidant capacity [62].

#### 6.1.2. Terpenoids as Modern Medicine

Beyond their historical role in traditional medicine, terpenoids have emerged as a promising class of bioactive compounds in modern drug discovery and therapeutic development. In recent years, increasing scientific attention has been directed toward exploring terpenoids as a source of novel pharmacological agents for the treatment of complex diseases (Table 1). A notable area of application is oncology, where several terpenoid derivatives have demonstrated significant anticancer efficacy. For example, paclitaxel, a diterpenoid originally isolated from the *Taxus brevifolia* (Pacific yew tree), has become a cornerstone chemotherapeutic used in the treatment of breast, ovarian, and lung cancers [63,64]. Terpenoids have also shown therapeutic potential in addressing other medical conditions, including neurological disorders, cardiovascular conditions, diabetes mellitus, and other infectious diseases. Notably, ginkgolides, a class of diterpenoids from *Ginkgo biloba*, have been investigated for their ability to improve cognitive performance in patients with Alzheimer’s disease [65]. Antimicrobial properties of terpenoids have gained renewed relevance in the fight against antibiotic resistance. Multiple studies have reported their efficacy against bacterial, fungal, and viral pathogens, suggesting their potential as alternative or complementary agents to combat drug-resistant microorganisms.

**Figure 2:**
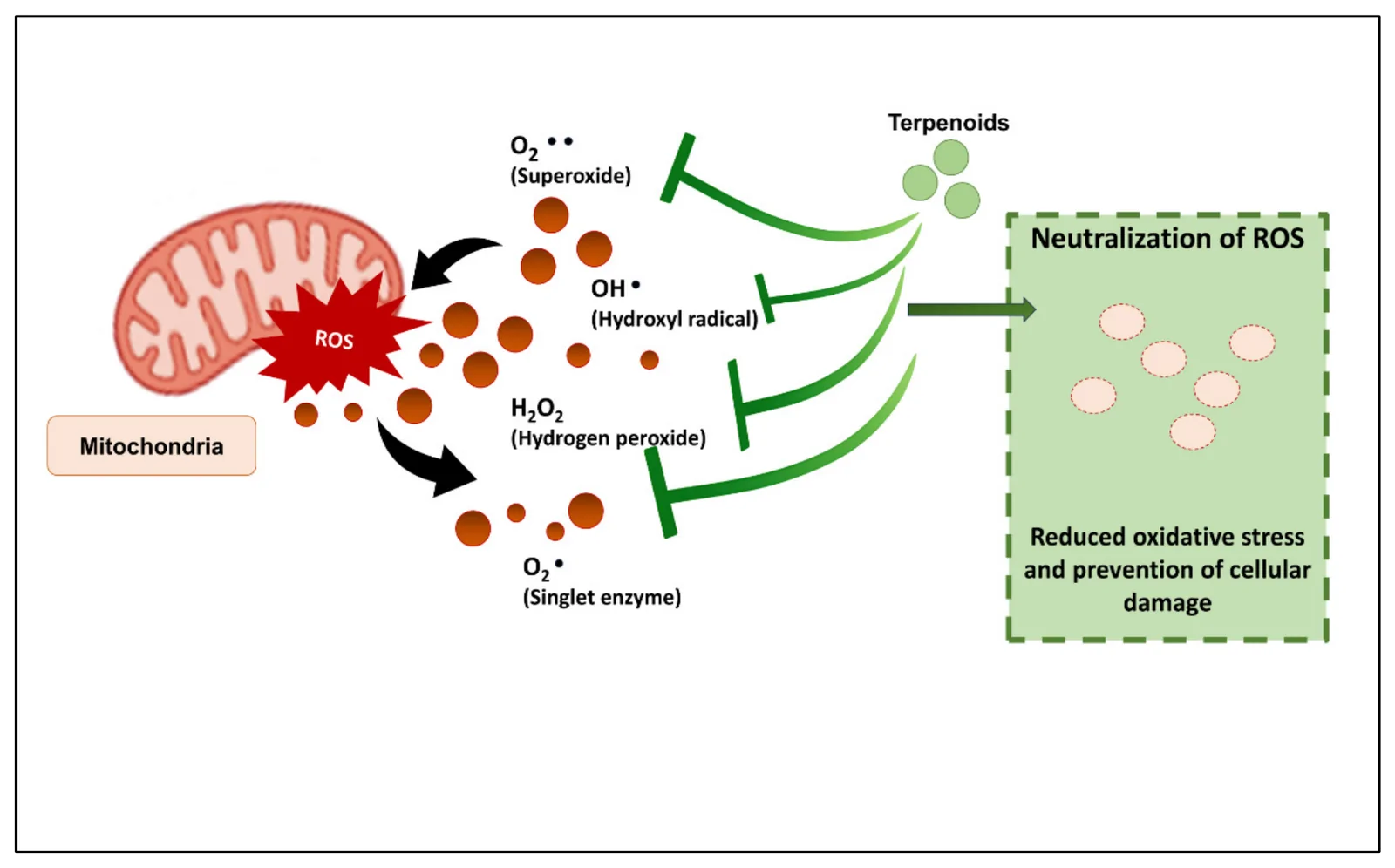
Antioxidant mechanism of terpenoids.

**Figure 3:**
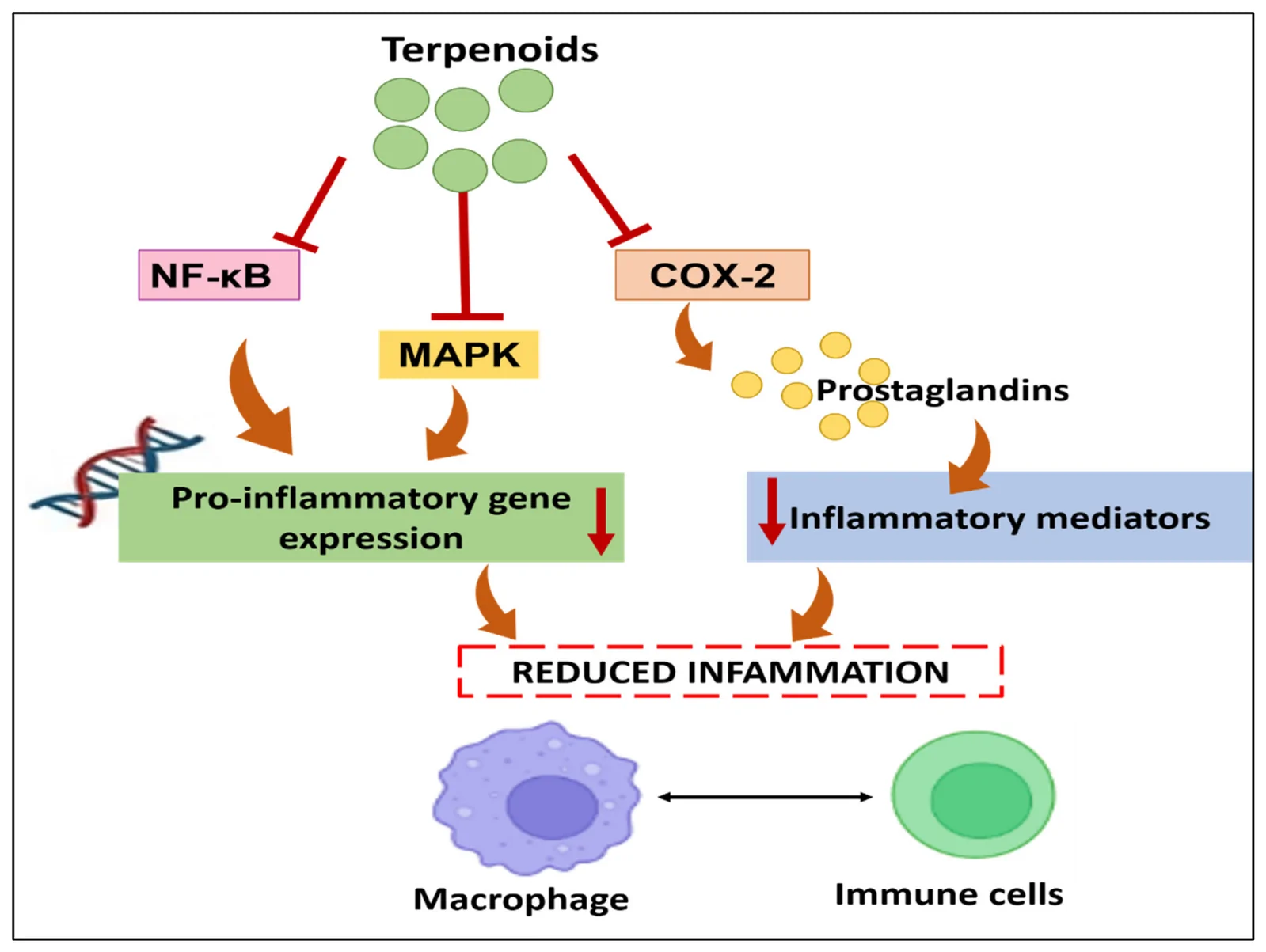
Anti-inflammatory action of terpenoids.

**Figure 4:**
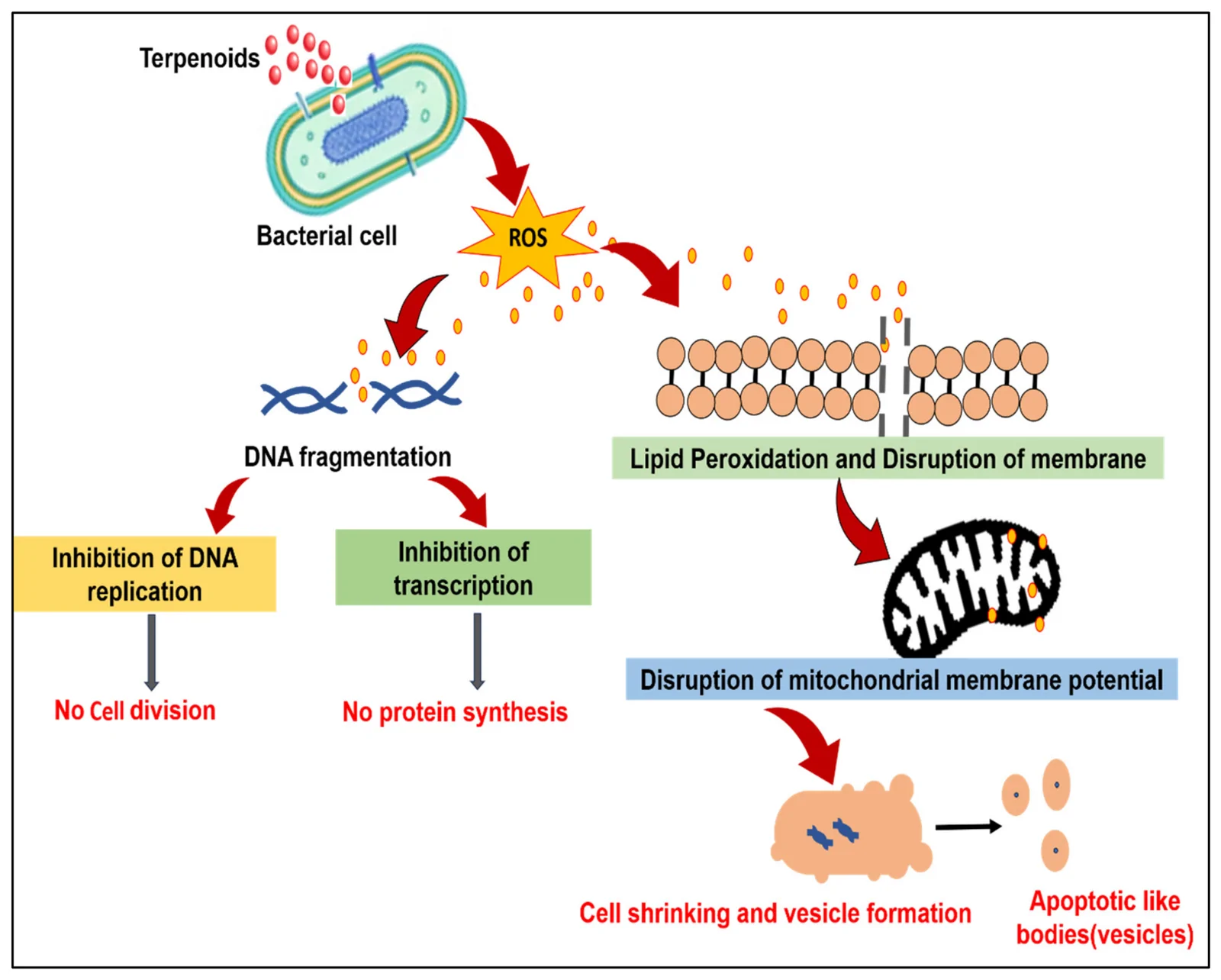
Antimicrobial action of terpenoids via DNA damage and apoptosis [83].

**Figure 5:**
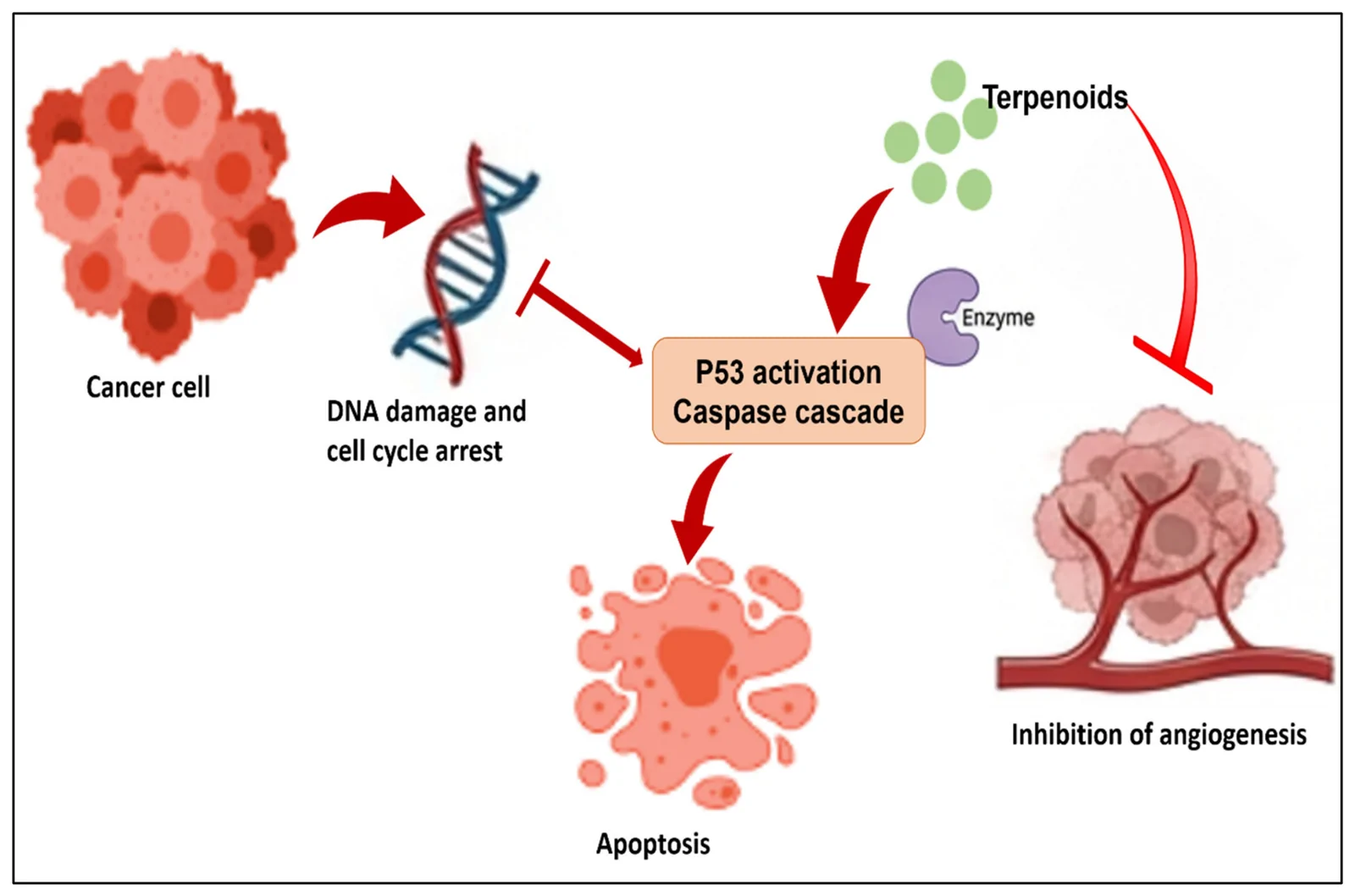
Terpenoid as anticancer agent.

- **Terpenoids as Antioxidants:** Antioxidants are bioactive molecules that mitigate oxidative stress by scavenging free radicals, thereby protecting cellular components from oxidative damage (Figure 2). They play a critical role in maintaining redox homeostasis and preventing degenerative processes. In biological systems, molecules such as glutathione, mycothiol, and superoxide dismutase play vital roles in mitigating oxidative stress damage [66]. The methanolic extract of *Commelina benghalensis* has been reported to contain triterpene (CB-1, CB-2 and CB-3) exhibiting significant antioxidant activity [67]. However, antioxidants play a crucial role in scavenging these harmful species, thereby preserving cellular integrity [68]. In a study on a rat model, limonene was shown to possess notable antioxidant potential that exerted a therapeutic effect against ulcerative colitis [69]. Among the various terpenoids tested, α-pinene demonstrated the highest reactive oxygen species (ROS) scavenging ability, suggesting its effectiveness in oxidative stress modulation [70]. Lycopene, a tetraterpenoid, is also recognized for its free radical-neutralizing capacity, cardioprotective benefits and inhibition of lipid antioxidation, making it valuable in preventing oxidative damage at the cellular and tissue level [71]. Marine terpenoids reduce oxidative stress by enhancing endogenous antioxidant defenses, including superoxide dismutase (SOD), catalase (CAT), glutathione peroxidase (GPx), and glutathione (GSH). By lowering reactive oxygen species (ROS) levels, they indirectly suppress oxidative reactions that encourage advanced glycation end product (AGE) formation. AGEs form when protein amino groups react with carbohydrate carbonyls or carbonyl/dicarbonyl compounds, producing Schiff bases, Amadori products, and ultimately AGEs [72,73]. Our research group recently reported the formation of AGEs extensively [74,75]. Additionally, terpenoids activate the Nrf2/HO-1 pathway and inhibit NF-κB signaling, thereby reducing oxidative stress and inflammation associated with AGE–RAGE interactions. This coordinated antioxidant and anti-inflammatory action helps interrupt the cycle between ROS generation and AGE accumulation [76].

- **Anti-Inflammatory Activity:** Inflammation, derived from the Latin term *inflammare*, meaning “to set on fire”, is a natural protective response of the immune system to infection, tissue injury, or other harmful stimuli (Figure 3) [77]. A number of bioactive terpenoids have demonstrated anti-inflammatory properties in experimental studies. Among them, citral, a major constituent of essential oils obtained from plants such as *Lippia alba* and *Cymbopogon citratus*, has shown significant anti-inflammatory activity in rodent models, highlighting its potential therapeutic value. Similarly, citronellal, a structurally related monoterpenoid found in several aromatic plants, has been reported to exhibit antinociceptive activity [78]. D-limonene has also attracted attention for its potential anti-inflammatory and stress-modulating effects, which may involve the regulation of inflammatory mediators and associated signaling pathways [79]. In addition, wood-derived terpenoids obtained from *Cunninghamia konishii* have demonstrated biological activities of potential relevance to the development of anti-inflammatory agents [80]. Furthermore, Araruna et al. [81] highlighted the potential of various terpenes and terpenoids to modulate intestinal inflammation, suggesting their possible application in the management of inflammatory gastrointestinal disorders. These compounds contribute to restoring gut homeostasis by reducing oxidative stress, enhancing intestinal barrier function, and addressing dysbacteriosis. Additionally, (-)-Linalool, a naturally occurring monoterpenoid enantiomer, has shown considerable anti-inflammatory properties. Its esterified derivative linalyl acetate also demonstrates both analgesic and inflammation-reducing effects [82].

- **Antimicrobial Activity:** Antimicrobial agents refer to substances capable of inhibiting the growth or causing the destruction of microorganisms, including bacteria, fungi, and protozoa. Several terpenoids, particularly monoterpenes, have been extensively studied for their antimicrobial activity due to their ability to interfere with microbial membrane integrity and function (Figure 4). These compounds exert their effects by disrupting the cell membrane, leading to an imbalance in membrane potential, interruption of respiratory processes, and leakage of intracellular components. For example, carvacrol and thymol, both terpenes, have demonstrated inhibitory and altering electrochemical gradients. A triterpenoid saponin, specifically 28-O-D-glucopyranosyl, 2-hydroxy-12-oleanene-3, has been reported to impair the antioxidant defense mechanisms of *Microcystis aeruginosa* cells by promoting oxidative membrane damage [83]. Terpenes from botanical sources, such as species from the Pinaceae family (e.g., *Pinus ponderosa*) and aromatic herbs like cumin, rosemary, thyme, clove, caraway, and sage, have shown considerable potential in mitigating microbial growth. *Cretan propolis* is also recognized for its terpenoid-rich antimicrobial profile [84]. In a comprehensive analysis, 52 different terpenoid compounds with confirmed antimicrobial activity were identified. These included essential oil constituents such as α-pinene (10.3%), α-cedrene (9.7%), aromadendrene (4.3%), β-caryophyllene (4.1%), and limonene (3.7%). Additionally, several esterified terpenoids contribute to microbial inhibition, including neryl acetate (11.6%), 2-methylcyclohexyl octanoate (4.9%), 2-methylcyclohexyl pentanoate (8.30%), and geranyl acetate (4.6%) [85]. A recent study isolated the strongest bactericidal fraction against E. faecalis from *Rosmarinus officinalis* essential oil, which included rich oxygenated monoterpenes such as myrtenol, verbenone, p-cymen-8-ol, γ-terpinen-7-al, carvone, and β-thujone [11].

- **Antiparasitic Agents:** Antiparasitic agents are chemical substances or medications designed to eliminate or suppress parasites, including protozoa, helminths (worms), and ectoparasites, which inhibit or invade host organisms and often lead to disease. These compounds function by targeting specific biochemical pathways crucial to parasite survival. A study has shown that terpenoids such as farnesol, nerolidol, limonene, linalool, and S-farnesylthiosalicylic acid (FTS) exert antimalarial properties against the intra-erythrocytic stages of *Plasmodium falciparum*, the parasite responsible for malaria [86]. Nerolidol interferes with the synthesis of the isoprenic side chain attached to the benzoquinone ring, while limonene blocks protein isoprenylation, an essential post-translational modification. Farnesol and linalool act by inhibiting intermediate synthesis within the isoprenoid biosynthesis pathway. These mechanisms likely disrupt parasite polyisoprenoid production by either competing with natural substrates or inhibiting isoprenic chain elongation [87]. Limonene has also been reported to inhibit the synthesis of dolichol and ubiquinone, which are both critical for parasite viability. S-farnesylthiosalicylic acid (FTS) treatment has been observed to suppress Ras signaling during the schizont stage of Plasmodium, halting parasite growth [88]. The rising prevalence of drug-resistant malaria strains has intensified the search for alternative therapies. Caryophyllene, pinene, limonene, and β-myrcene have shown promising antiplasmodial activity [89]. Among the terpenoid-based antimalarials, one of the most notable drugs is artemisinin, which is a sesquiterpene lactone that is isolated from *Artemisia annua* L. The key feature of artemisinin is the presence of an endoperoxide bridge, which is believed to be responsible for its antiplasmodial action and has contributed to its importance in the development of antimalarial drugs through natural products. Some of its derivatives, such as artesunate, artemether, and arteether, have been synthesized in order to enhance their pharmacological effects and medicinal use. Artemisinin derivatives are widely used in combination with other antimalarial drugs in ACTs for increasing effectiveness and minimizing the chances of resistance [90]. Additionally, the sesquiterpene nerolidol has shown antiplasmodial activity in laboratory experiments.
- **Anticancer Agents:** Cancer remains one of the most pressing global health challenges, accounting for millions of new cases worldwide each year. Natural products and their derivatives have played an important role in the development of several clinically effective anticancer agents (Figure 5) [91]. Among naturally occurring compounds, dietary monoterpenes, particularly D-limonene and perillyl alcohol, have attracted considerable attention as potential chemopreventive and anticancer agents. Preclinical studies have demonstrated their activity against a range of malignancies, including cancers of the skin, lung, liver, breast, prostate, stomach, pancreas, and colon, although their efficacy varies considerably depending on the experimental model and treatment conditions [92]. Farnesylthiosalicylic acid (FTS) has also been reported to inhibit the growth of human pancreatic tumors by interfering with Ras membrane association and promoting its degradation [87]. In addition, several classes of terpenoids have been extensively investigated for their potential antitumor properties. These include sesquiterpene lactones, such as parthenolide, costunolide, helenalin, and ambrosin; diterpenoids, such as triptolide; and other terpenoid compounds, including crocetin, ursolic acid, and lupeol. These compounds can modulate multiple cellular signaling pathways and molecular targets associated with tumor initiation and progression, cell proliferation, oxidative stress, cell-cycle regulation, and apoptosis.

β-elemene, a sesquiterpene originally identified from plants used in traditional Chinese medicine, has demonstrated broad-spectrum anticancer activity and has been investigated for its potential application against several malignancies, including brain, gastrointestinal, and lung tumors. Its reported anticancer mechanisms include the induction of apoptosis, inhibition of tumor-cell proliferation, and modulation of cancer-associated signaling pathways [93].

Furthermore, naturally occurring compounds such as β-caryophyllene have attracted interest because of their potential biological and anticancer activities. However, the therapeutic relevance of such compounds should be distinguished from their regulatory approval as food additives or flavoring agents. Overall, terpenoids represent a structurally diverse source of bioactive molecules with considerable potential for anticancer drug discovery and development, although further pharmacokinetic, toxicological, and well-designed clinical studies are required to establish their therapeutic efficacy and safety.

**Figure 6:**
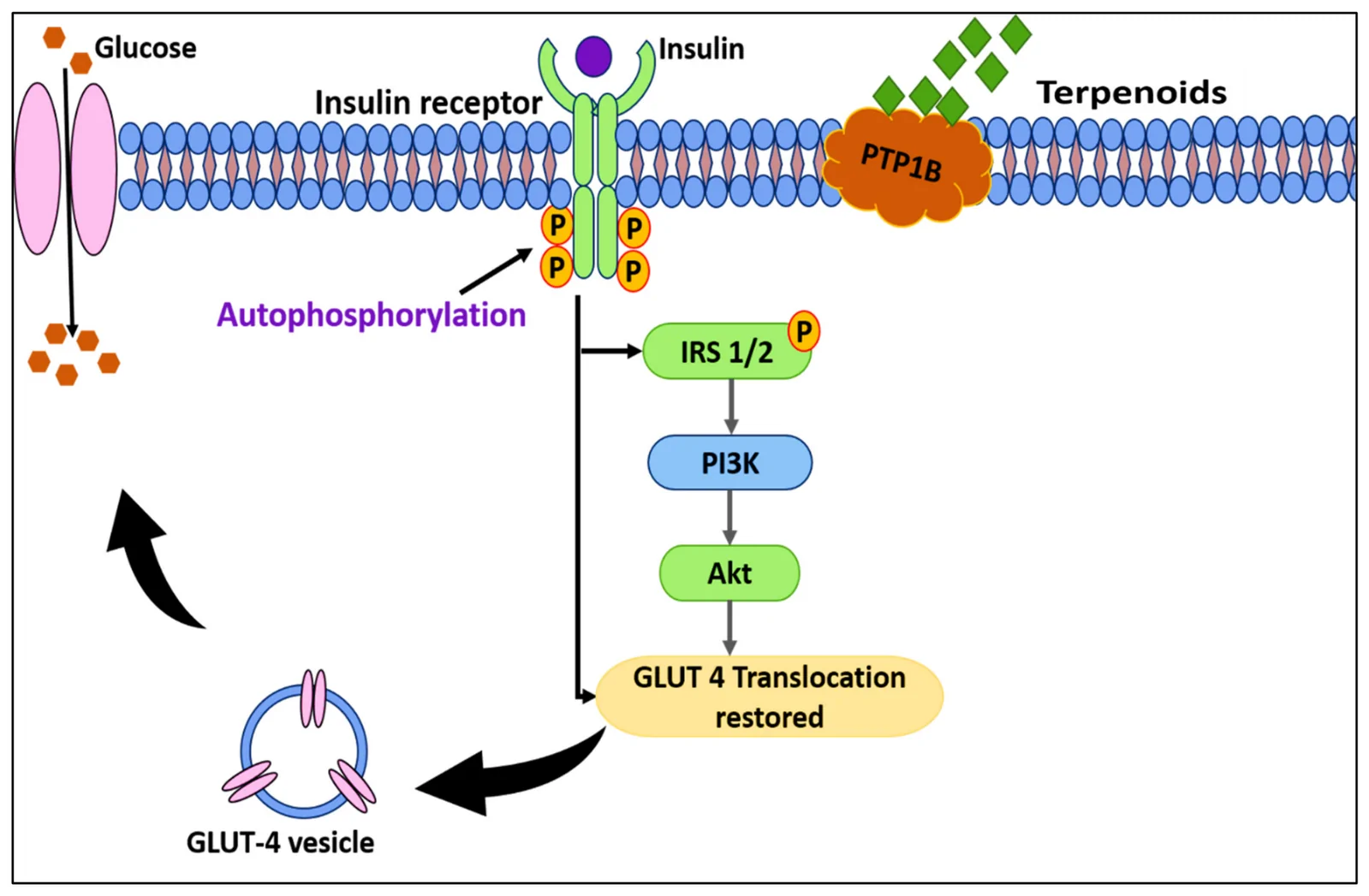
Terpenoid as an antidiabetic agent targeting PTP1B, one of the negative regulators of the insulin signaling pathway.

- **Antidiabetic Agents:** Type 2 diabetes mellitus is a chronic metabolic condition characterized by impaired insulin secretion in its early stages, leading to elevated blood glucose levels over time [4]. Among natural compounds, stevioside, a diterpene glycoside isolated from the leaves of *Stevia rebaudiana*, has shown promising therapeutic effects (Figure 6). It enhances insulin secretion, modulates glucagon levels, and effectively reduces hyperglycemia, highlighting its potential role in diabetes management [94]. Several triterpenes, such as gymnemic acid IV, maslinic acid, arjunolic acid, and platycodin D, have demonstrated strong antidiabetic activities through various biological mechanisms [95]. Notably, terpenoids extracted from *Cleome droserifolia* have been reported to improve basal glucose uptake in in vitro models using C2C12 skeletal muscle cells and 3T3L1 adipocytes. Furthermore, two insulin-sensitizing triterpenes have been isolated from bitter melon, which, in the presence of insulin and the proinflammatory cytokine TNF-α, significantly enhanced the phosphorylation of the key signaling molecules IRS1 and Akt (protein kinase B), which play a pivotal role in glucose metabolism. Ursolic acid, a triterpenoid found in the leaves of *Rosmarinus officinalis*, also exhibits pronounced antihyperglycemic properties [96].

Plant-derived terpenoids generally have been recognized for their ability to inhibit Protein tyrosine phosphate-1B (PTP1B), a negative regulator of insulin signaling. For instance, diterpenes from the coral *Sarcophyton trocheliophorum* and triterpenoids and triterpenoids from Cedrela odorata have been found to significantly suppress PTP1B enzyme activity. Despite the well-documented bioactivities of terpenoids in preclinical settings, the number of clinical trials reaching advanced phases remains limited. However, a few notable studies highlight the translational potential of selected terpenoids. Terpenoids also demonstrate protective effects in diabetes by targeting key enzymes involved in glycation and polyol pathway activity. They hinder α-glucosidase and aldose reductase, thereby reducing the formation of advanced glycation end products (AGEs) and limiting sorbitol accumulation associated with neuronal damage. Inhibition of α-glucosidase helps control postprandial glucose levels, decreasing the availability of high glucose for non-enzymatic glycation. Concurrently, suppression of aldose reductase reduces polyol pathway flux, minimizing osmotic imbalance and oxidative stress that contribute to AGE formation [76]. Table 2 summarizes some key recent clinical trials conducted on terpenoid-based interventions, particularly targeting metabolic disorders, inflammation and cancer.

- **Miscellaneous:** The antiviral potential of terpenoids remains incompletely explored, highlighting the need for further research to identify natural compounds with potent and selective antiviral activity. Ideally, such compounds should effectively inhibit viral replication while exerting minimal adverse effects on healthy host cells. The search for naturally derived antiviral agents has led to the identification of several promising terpenoids, including isoborneol. Monoterpenes such as borneol and 1,8-cineole have also demonstrated activity against herpes simplex virus type 1 (HSV-1), suggesting their potential as lead compounds for the development of novel antiviral agents [101].
- Insects have a substantial impact on human health and agricultural productivity. Although synthetic pesticides are widely used for insect control, their extensive and inappropriate use has contributed to the emergence of pesticide-resistant insect populations, potential risks to human health, contamination of food products, and environmental pollution. As potentially safer and more sustainable alternatives, medicinal plants and their essential oils have attracted considerable attention as sources of natural insecticidal compounds. Essential oils rich in terpenes and terpenoids, including 1,8-cineole, β-ionone, carvacrol, eugenol, linalool, and limonene, have demonstrated promising insecticidal and repellent activities. These natural compounds have shown activity against a range of medically and agriculturally important insects and arthropods, including lice, cockroaches, and triatomine bugs [102].
- The development of effective strategies for the prevention and management of cardiovascular diseases remains another major focus of biomedical research (Figure 3). Among the terpenoids investigated for cardiovascular effects, tanshinone IIA, a bioactive diterpenoid derived from the roots and rhizomes of *Salvia miltiorrhiza* Bunge, has received considerable attention. Experimental studies suggest that tanshinone IIA may inhibit atherosclerotic plaque formation and attenuate cardiac hypertrophy and myocardial injury [103]. Another bioactive compound, hinokitiol, has been reported to protect cardiomyocytes against oxidative stress-related injury through modulation of GSK-3β-associated signaling and regulation of the balance between apoptosis and autophagy [104]. Carvacrol, which possesses antioxidant and anti-apoptotic properties, has also demonstrated cardioprotective effects in experimental models of myocardial ischemia–reperfusion (I/R) injury. These effects have been associated with modulation of the Akt/eNOS and ERK/MAPK signaling pathways [105].
- Tuberculosis (TB) continues to represent a major global health challenge, emphasizing the need for new therapeutic agents, particularly in the context of drug-resistant *Mycobacterium tuberculosis*. Several naturally occurring terpenoids have therefore been investigated as potential sources of antitubercular compounds. Diterpenoids such as isosteviol and some of its derivatives, as well as triterpenoids, including betulinic, oleanolic, and ursolic acids, have demonstrated inhibitory activity against *M. tuberculosis* in experimental studies, with computational approaches such as molecular docking providing additional insights into their potential molecular interactions. Other naturally occurring terpenoids, including geranylgeraniol, phytol, escobarine A, escobarine B, furanoditerpenes, salasol A, germacrane-type compounds, and alantolactone, have also been investigated for potential antitubercular activity [106]. Collectively, these findings highlight the chemical diversity of terpenoids and their potential as sources of lead compounds for antimicrobial and therapeutic development, although further mechanistic, pharmacokinetic, toxicological, and clinical investigations are required to establish their therapeutic relevance.

### 6.2. Terpenoids as Agents for Promoting Longevity

Geroprotectors represent a rapidly emerging and fascinating field aimed at identifying pharmacological agents or interventions capable of slowing down aging and extending lifespan. Among natural bioactive compounds, terpenoids have shown considerable promise in this domain due to their antioxidant and anti-inflammatory effects [107]. These compounds modulate key biological pathways associated with oxidative stress, inflammation, and cellular senescence, which are fundamental hallmarks of the aging process. The scientific validation of any compound as a geroprotector remains a complex and evolving process. An interesting example involves limonene, a monoterpene found in numerous aromatic plants. In *Ceratitis capitata* (Mediterranean fruit fly), low-dose exposure to limonene produced a hermetic effect, where sublethal concentrations (LD20 at 3.47 nL per male and 12.26 nL per female) led to lifespan extension and enhanced female fertility, particularly under protein-deficient dietary conditions. In contrast, higher doses (LD90 at 39.74 nL per male and 75.51 nL per female) were toxic [108]. Similar studies on *Drosophila melanogaster* reported that every low concentration of limonene (0.011 and 0.046 mM) was associated with a significant increase in both median and maximum lifespan [109]. Research using *Caenorhabditis elegans* further supports the potential of terpenoids as geroprotectants. Dehydroabietic acid extended lifespan while also reducing lipofuscin accumulation and fibrosis development [110]. Moreover, treatment with carnosol and carnosic acid led to notable improvements in both average and maximum lifespan in a nematode model [111].

### 6.3. Terpenoid Utility in Agriculture

Terpenoids have gained considerable attention in modern agriculture as sustainable alternatives to pesticides and synthetic plant growth regulators. Growing concerns over the environmental and health-related consequences of synthetic agrochemicals have accelerated the shift towards more ecofriendly and biologically safe options, with terpenoids offering a compelling solution [8]. Terpenoid-based pesticides have demonstrated insecticidal, fungicidal, and herbicidal properties, like neem oil, which contains azadirachtin, a terpenoid compound derived from *Azadirachta indica*. It functions as both an insect growth regulator and repellent effective against numerous agriculture pests [112]. In addition to their pesticidal roles, terpenoids act as natural plant growth regulators, influencing essential physiological processes, such as seed germination, root elongation, and flower induction; for example, Gibberellins, a group of diterpenoid plant hormones, are widely employed to improve agricultural productivity by promoting traits like increased fruit size and shortened flowering periods [113]. Volatile terpenes are involved in complex plant signaling pathways. They play vital roles in attracting pollinators, deterring herbivores, and mediating plant–biotic environment interactions, thereby contributing to plant survival and reproductive success [114]. Additionally, through chemical modifications, a wide array of functional derivatives can be synthesized using β-pinene as the starting molecule. These derivatives have been shown to possess diverse pharmacological and agrochemical activities, including applications as insecticides, fungicides, repellents, antifeedants, and pesticide synergists [93].

### 6.4. Terpenoids in Industrial Formulations

Terpenoids are widely used across various industrial sectors, particularly in the production of fragrances, flavors, and a range of consumer products. These compounds contribute to the characteristic aromas and flavors of many everyday products, including perfumes, cosmetics, food products, and beverages [115]. Terpenoid-rich essential oils extracted from plants such as *Rosa damascena* (rose), *Lavandula* spp. (lavender), and *Citrus* spp. (citrus fruits) are widely used in the fragrance industry because of their distinctive and complex aromatic profiles [116].

Terpenoids also play an important role in determining the flavor profiles of food and beverage products. For example, limonene, a monoterpene abundant in citrus fruits, provides a characteristic citrus flavor and is widely used as a flavoring agent in various foods and beverages [117]. Similarly, menthol, primarily obtained from peppermint, is an important flavoring and cooling agent used in confectionery products, oral hygiene products, and pharmaceutical formulations [118]. Beyond their aromatic and flavoring properties, terpenoids have also attracted considerable interest for the development of environmentally friendly products, including biodegradable materials, biofuels, and other sustainable alternatives [9]. Table 3 summarizes commercially available terpenoid-based products and their diverse applications.

Despite their considerable commercial and therapeutic potential, the large-scale industrial utilization of terpenoids faces several challenges. Many terpenoids exhibit poor aqueous solubility, limited stability and bioavailability, and susceptibility to degradation during processing and storage. Furthermore, batch-to-batch variations in naturally derived terpenoids, together with difficulties in maintaining consistent composition and quality, can complicate product standardization. These limitations are particularly important for pharmaceutical formulations, where reproducibility, stability, purity, and standardized concentrations of active compounds are essential for ensuring consistent product quality and therapeutic performance.

Isoprenoids, a class of terpenoids, have drawn significant interest due to their diverse industrial potential, ranging from personal care and household fragrances to biofuel, food ingredients and therapeutics. Among these, β-elemene, a terpene compound prominent in koji fermentation, plays a dual role in Chinese baijiu: it enhances the beverage’s characteristic flavor and also serves as a bioactive health-promoting molecule with anticancer, anti-inflammatory, and antibacterial properties. Another notable compound, limonene (1-methyl-4-isopropyl-cyclohexene), commonly found in citrus, vegetables and spices, contributes not only a pleasant lemon-like aroma but also functional benefits in alcoholic beverages such as beer and baiju, where it imparts a mild hop scent and bitterness [115]. In addition, farnesol is responsible for the signature fragrance of bell orchid and is extensively used in cosmetics and alcohol-based beverages, including baiju, for its aromatic appeal. Likewise, β-caryophyllane is also present in fermented wine, raw wine, fermented grains, and Daqu medicinal baijiu, contributing both flavor and health-related attributes [129]. Pinene, another vital terpene, serves as a key precursor for the synthesis of several fragrance and flavor compounds, including terpineol, linalool, and sandalwood-like aroma compounds [130]. Industrially, α-pinene-derived terpene resin is widely used in paints and printing inks [131]. Pinene derivatives also serve as plasticizers, wood preservatives, epoxy resin curing agents, adhesives, and rubber-processing additives, highlighting their versatility in industrial applications. Lastly, geraniol, an acyclic monoterpene found in roses and peonies, contributes significantly to their fragrance and is extensively used in the food, fragrance, and cosmetic industries due to its floral, sweet aroma and bifunctional properties [132].

Aromatherapy is a complementary therapeutic practice that uses volatile aromatic compounds found in essential oils, particularly terpenoids, to support mental and physical well-being. In this practice, essential oils are either inhaled through diffusion or applied topically, often in combination with massage or relaxation therapies to enhance their effects [133]. Notable terpenoids such as linalool, found in lavender and bergamot oils, and α-pinene, present in pine and rosemary oils, are known for their potential calming and stress-relieving effects [134]. These compounds interact with olfactory receptors and, in some cases, may influence central nervous system pathways by modulating neurotransmitter activity, thereby potentially improving mood and promoting relaxation. In addition to their psychological effects, terpenoid-containing essential oils are also used to help manage physical symptoms such as headaches, insomnia, and muscle pain. For instance, peppermint oil, which is rich in menthol, is commonly applied topically to the temples for the relief of tension-type headaches and migraines [135].

## 7. Current Trends and Challenges

Terpenoids, which are essential for human health and medicine, are structurally diverse but difficult to synthesize, which impedes their mass production. Their industrial application is restricted by their high costs and inadequate yields resulting from natural extraction. Multiple stereoisomers often arise during chemical synthesis, raising concerns regarding potential health effects. Despite having numerous therapeutic uses, the range of clinical applications is restricted due to the limited bioavailability, poor water solubility, lack of absorption facilitation, and volatile terpenoid oil status [136]. In addition, therapeutic administration of terpenoids has been associated with side effects such as bleeding, fever, and local pain. Limonene, with notable antibacterial and therapeutic properties, is derived from multiple botanical sources and holds substantial economic and pharmaceutical potential. However, its commercial utilization remains limited due to challenges such as low extraction yields, suboptimal purity, and inadequate safety assurance during natural sourcing. Research in clinical medicine is sparse, despite the fact that it has been extensively explored in the food industry as a condiment, antioxidant, and preservative. However, recent pharmacological and toxicological evaluations suggest that limonene and its enriched formulations may find promising applications in the pharmaceutical sector. Additionally, β-caryophyllene is chemically unstable; its double bond is prone to oxidation into peroxidation upon air exposure, potentially leading to skin irritation and allergic reactions [108]. This restricts the therapeutic use of β-caryophyllene; consequently, technical strategies to prevent its oxidative degradation could pave the way for its safe and effective clinical application.

In order to resolve this issue, sophisticated techniques such as chemical engineering and enzymatic catalysis provide precise control over terpenoid levels. Advancements in isomer separation techniques have enabled the production of pure terpenoid compounds, thereby mitigating health-related concerns associated with isomeric impurities. Despite the obstacles associated with synthetic and natural sourcing, engineered industrial cell factories offer safe and economically viable terpenoid production through strategies such as metabolic engineering and the elimination of feedback pathways [53]. Although a large number of terpenes have been identified, significant gaps remain in our understanding of their chemical synthesis and biological biosynthesis routes. Terpenoids are frequently detected in trace quantities in natural sources, necessitating the implementation of effective isolation and purification techniques. Conventional extraction methods are often labor-intensive and inefficient, leading to challenges in obtaining terpenoids of sufficient purity for industrial or pharmaceutical use, like taxol, an anticancer drug. In order to thoroughly investigate the potential of terpenes in medicine and other applications and to address these gaps, additional research is required [137]. The strain complexity optimization and metabolic pathways, as well as tasks associated with substrate availabilities, scale-up difficulties, and product toxicity, are among the limitations of bioprocesses for terpenoid production.

Furthermore, the ubiquitous industrial application of bioprocess-based terpenoid production is further restricted by stringent regulatory compliance and downstream processing complexities. While there have been some methods proposed for the complete synthesis of taxol [138], such multi-step approaches typically yield only relatively small amounts of the final product, insufficient to satisfy the significant pharmaceutical demand [139,140]. Besides the challenges in purification, terpenoids are prone to degradation upon exposure to heat, light, and oxygen, potentially resulting in the loss of their beneficial properties. Formulating products that necessitate terpenoids as active constituents is complicated by this instability. Additionally, terpenoids, particularly those employed in pharmaceuticals and nutraceuticals, may encounter obstacles associated with their bioavailability, which refers to the rate and extent of their absorption and subsequent offering in the circulation [10]. Innovative formulation techniques are frequently necessary to improve the bioavailability of terpenoids.

Recent advances in extraction technologies, including molecular distillation and supercritical fluid extraction, have significantly improved the yield and purity of terpenoids, making them more suitable for applications with stringent industrial quality requirements [140]. Nevertheless, the development of fully bioengineered biosynthetic pathways offers a promising and potentially more efficient approach for the sustainable production of terpenoids and their analogues. Terpenoid production can be further enhanced by eliminating negative-feedback regulation within precursor biosynthetic pathways, thereby enabling the development of engineered strains with substantially improved productivity. In addition to metabolic engineering, optimization of bioprocess parameters, including culture medium composition, pH, temperature, aeration, agitation, and fermentation conditions, is essential for maximizing terpenoid yields [141]. Advances in process automation, real-time monitoring, and innovative bioreactor design now enable more precise control of fermentation conditions and improved process reproducibility. Collectively, these technological developments can facilitate the efficient scale-up of terpenoid production and support their transition from laboratory-scale biosynthesis to commercially viable industrial applications [142].

Terpenes are generally regarded as safe in limited quantities, but research has revealed their toxic nature through in vitro and in vivo studies when focusing on safety concerns [143] (Table 4). Lipid-based formulations and delivery systems have demonstrated efficacy in protecting terpenoids from degradation while improving their absorption and bioavailability [144]. However, animal studies have raised safety concerns at some point. α-Terpinene (intraperitoneally) has been shown to induce oxidative, cytotoxic, and genotoxic effects. Epidermal contact (skin) with terpenoids may lead to allergic reactions, while inhalation may trigger respiratory irritation, particularly in sensitive individuals. Although the ingestion of terpenes in small quantities is generally safe, concentrated forms (such as essential oils) may induce toxic effects, such as gastrointestinal inflammation. To reduce these risks, safe usage trails including dilution, adequate ventilation, and patch testing must be strictly followed. Several strategies can mitigate terpene toxicity, such as α-terpinene toxicity, including dose modulation, appropriate route selection, and advanced formulation techniques, like nanocarrier encapsulation or sustained release systems, as suggested by Lima et al. [145]. Additionally, conjugating terpenes with synergistic or protective compounds may further improve their safety profiles [146]. It is imperative to conduct severe preclinical studies that concentrate on the search of precise mechanisms and the establishment of safe dosage thresholds. This finding is reinforced by the absence of treatment-related impacts on rats’ overall health, behavior, clinical pathology indicators, and organ evaluations, indicating a positive safety profile [147]. Moreover, the growing inclination towards natural and holistic health practices is evident in the observation that terpenoids derived from natural sources often have fewer side effects in a dose-dependent manner compared to synthetic medications. While bioavailability remains a key challenge, current research efforts are focused on developing advanced formulations and delivery systems that can improve absorption and efficacy, thereby maximizing the therapeutic value of terpenoids in diabetes care.

## 8. Strategies to Enhance Terpenoid Production

Terpenoids are synthesized in relatively low quantities in nature, which poses a limitation on their large-scale utilization across various industries. Given their diverse pharmacological and industrial applications, the demand for methods that boost terpenoid biosynthesis has significantly increased. To address the naturally low yields of terpenoids in biological systems, various enhancement techniques have been employed. These methods vary in complexity, cost, efficiency, and scalability. Table 5 below compares the most prominent strategies. Among the range of strategies that have been developed to enhance production, the most effective method is the use of elicitor substances that trigger plant defense and metabolic responses. These can be broadly categorized into biotic (originating from living organisms) and abiotic (non-living) types, both of which activate secondary metabolic pathways, thereby stimulating the biosynthesis of terpenoids [150]. Biotic elicitors such as salicylic acid and jasmonates (plant signaling molecules) are frequently employed to elevate terpenoids. For instance, certain bacterial elicitors have been shown to enhance terpenoid production in Ginkgo biloba, while cultures of adventitious roots in *Andrographis paniculata* exhibited increased andrographolide accumulation under elicitor treatment [151]. In another study, adventitious roots of *Peritassa campestris* (Celastraceae) were generated from seed cotyledons using a semisolid woody plant medium supplemented with polyvinylpyrrolidone (PVP), indole-3-butyric acid (IBA), and sucrose. After seven days of incubation, the culture yielded a maximum concentration of maytenin, a quinone methide-class triterpenoid, of 972 µg/g dry weight (DW), which was 5.55 times higher than the levels observed in greenhouse-grown seedlings [152]. Biotic elicitation has also proven valuable for the commercial production of anticancer agents such as paclitaxel. For example, *Taxus chinensis* suspension cells co-cultured with its endophytic fungus *fusarium mairei* in a 20-L bioreactor produced 25.6 mg/L of paclitaxel within 15 days, a productivity rate that is 38 times higher than that of control cultures without fungal elicitation (0.68 mg/L), averaging 1.71 mg/L per day [153].

Abiotic stress agents, particularly heavy metal salts, have demonstrated considerable potential in boosting terpenoid production in plant tissue cultures. For instance, cadmium chloride (CdCl2) treatment significantly enhanced andrographolide accumulation in a suspension culture of Andrographis paniculata. In *Panax ginseng* cells, vanadate exposure has been found to upregulate several genes involved in terpenoid biosynthesis, thereby promoting the accumulation of ginsenoside triterpenoid compounds. In 2018, Lu et al. [159] further substantiated the role of abiotic elicitors in increasing ginsenoside levels in a suspension culture of *P*. *ginseng*. To explore the optimal conditions for stimulating terpenoid biosynthesis, Guo et al. [160] evaluated the effects of various heavy metal salts, including NaVO3, NH4VO3, NiSO4, VOSO4, CuSO4, and MnSO4; among these vanadates, NaVO3 proved to be the most effective at enhancing total ginsenoside content (Rg1 + Re + Rb1 + Rc + Rd), reaching a peak concentration of 5.6 mg/g DW weight on day 14 following 4 days of treatment. Mechanistic studies revealed that this vanadate not only triggered endogenous jasmonic acid (JA) biosynthesis but also significantly elevated the transcription of key pathway genes, such as squalene synthase (SQS), squalene epoxidase (SE), and dammarenediol-II synthase (DS). Overall, these elicitors offer effective strategies for enhancing the production of medicinal terpenoids in plant cell cultures.

Furthermore, compartmentalization strategies for efficient terpenoid biosynthesis involve the targeted engineering of specific cellular organelles (Table 6). These include expanding the endoplasmic reticulum (ER) to enhance enzyme synthesis and processing, redirecting enzymes to lipid droplets (LDs) for localized concentration and efficient product formation, and employing peroxisomes as both storage and biosynthesis sites for terpenoids. Mitochondrial targeting helps exploit high acetyl-CoA availability and controlled ATP use, while plasma membrane modifications allow for spatial enzyme substrate proximity. Such compartment-level optimizations provide a robust platform for improving terpenoid yields through metabolic engineering and synthetic biology approaches.

Moreover, protein engineering strategies, like including directed evolution and rational design, play a pivotal role in enhancing enzyme performance within terpenoid biosynthetic pathways. Despite the potential, obstacles such as enzyme promiscuity and complex cyclization patterns continue to hinder rational enzyme optimization, necessitating deeper mechanistic insights. To complement these strategies, dynamic control systems, like quorum sensing, have emerged as innovative tools for regulating gene expression in real time based on intracellular or environmental cues. Such regulatory tools allow for the precise tuning of metabolic flux, thereby improving the yields of target terpenoids in engineered microbial platforms [173].

Recent developments in genome editing, particularly CRISPR-based technologies, have transformed molecular biology by enabling precise and targeted modifications to DNA sequences with living cells and organisms. In addition to CRISPR-Cas systems, tools like zinc-finger nucleases (ZFNs) and transcription activator-like effector nucleases (TALENs) have also been used to explore and implement genomic edits. A derivative system known as CRISPR interference (CRISPRi), or catalytically inactive Cas9 (dCas9), has enabled the fine-tuned regulation of gene expression without inducing permanent genomic changes. This is achieved by mutating the nuclease domains of Cas9 while preserving its DNA binding capacity, thereby enabling gene repression or activation with high specificity (Table 7). Such regulatory control has been effectively applied in pathways like terpenoid biosynthesis [174].

In synthetic biology, the design of synthetic promoters and ribosome binding sites (RBSs) plays a vital role in controlling transcription and translation rates, thereby enabling the precise regulation of biosynthetic gene clusters. By selecting promoter and RBS elements with predictable strength and behavior, researchers can systematically optimize pathway output for target compound production. Shukal et al. [175] utilized CRISPR-Cas9 to knockdown the LdhA gene part of a competing pathway leading to elevated acetyl-CoA levels and enhanced terpenoid yields. Similarly, Liu et al. [176] improved pyruvate and glyceraldehyde-3-phosphate (G3P) availability by overexpressing GalP and disrupting PtsHLcrr using CRISPR, thereby creating a metabolically superior host for terpenoid biosynthesis. Genes of the MVA pathway, which are often absent in native bacterial systems, are frequently introduced exogenously to reinforce terpenoid precursor supply. Such core pathway modifications remain essential strategies for maximizing terpenoid production in microbial cell factories.

**Table 7:** Applications of CRISPR-Cas9-based engineering for heterologous production of terpenoids.

Compound	Strain	Integrated Genes	Gene Origin	Increased Production	Ref.
Amorphadiene	*B. subtilis*	GFP-ADS, FPPS	Plant origin	Increased by 1.4-fold	[177]
Amorphadiene	*B. subtilis*	GFP-ADS, FPPS and overexpression of MEP pathway genes in plasmid	Plant origin	Increased by 20.0-fold	[178]
Squalene	*C. glutamicum*	SQS and high-throughput fermentation	Microbial origin	Increased by 5.0-fold	[179]
*β*-carotene	*E. coli*	Almgs	Microbial origin	Increased by 1.4-fold	[180]
Zeaxanthin		CrtZ		Increased by 1.3-fold	[181]
Astaxanthin		CrtW, CrtZ		Increased by 1.3-fold	[182]
Crocin	*E. coli* BL21 (DE3)	CCD, ALD	Plant and microbial origin	Increased by 3.6-fold	[183]
Steviol	*E. coli* MG1655	GGPPS, CPS, KS	Microbial origin	Increased by 2.5-fold	[184]
Bisabolene	*E. coli* DH1	HMGS, HMGR, MK, PMK, PMD, IDI, FPPS, AgBIS	Plant and microbial origin	Increased by 5.0-fold	[185]

Recent progress in computational biology has introduced machine learning as a powerful tool to predict promoter sequences and optimize regulatory elements for improved biochemical synthesis [186]. The data-driven models facilitate the rational design of promoters, enhancing gene expression control and maximizing metabolic activity. Cellular tolerance engineering also plays a vital role in boosting terpenoid production by minimizing the toxic effects of pathway intermediates. Efflux pumps or transporters assist in expelling harmful compounds from the cell, thereby reducing feedback inhibition and preventing cytotoxicity. To further enhance microbial tolerance, strategies such as inducible or tightly regulated promoter systems and adaptive evolution have been employed to maintain cellular health under production stress [187]. Chromosomal integration provides a more consistent and stable alternative to plasmid-based gene expression systems. Through approaches like site-specific recombination and homologous recombination, biosynthetic pathway genes can be stably integrated into the host genome, ensuring long-term expression without plasmid-associated limitations. Another effective strategy involves modularizing synthetic pathways, whereby large metabolic routes are divided into manageable units for individual optimization. This modular approach allows for the fine tuning of each segment, balancing precursor flux and improving overall pathway efficiency, thereby enhancing the yields of target terpenoids and other valuable compounds [187].

Additionally, multiple genetic engineering approaches have been adopted to elevate the production of pharmaceutical terpenoids in plants. These include the homologous overexpression of native genes, the co-expression of multiple biosynthetic genes, and the ectopic expression of genes from heterologous sources. In *Panax ginseng*, for example, the overexpression of genes such as PgSQS1 and PgHMGR has been shown to significantly boost ginsenoside accumulation [188]. Likewise, in *Salvia miltiorrhiza*, co-expression of SmHMGR and SmGGDS has led to the enhanced production of tanshinones, which are valuable diterpenoid compounds. Additionally, introducing terpenoid synthase or cyclase genes from other species into medicinal plants has proven effective in generating novel or increased quantities of pharmaceutical terpenoids. All these methods are used to enhance the production of terpenoids to make them more convenient and effective for industrial use. This can aid in commercializing natural source medicines (nutraceuticals) for use rather than synthetic ones, contributing to the production of less toxic and more effective drugs.

## 9. Strategies to Enhance Bioavailability of Terpenoids

**Figure 7:**
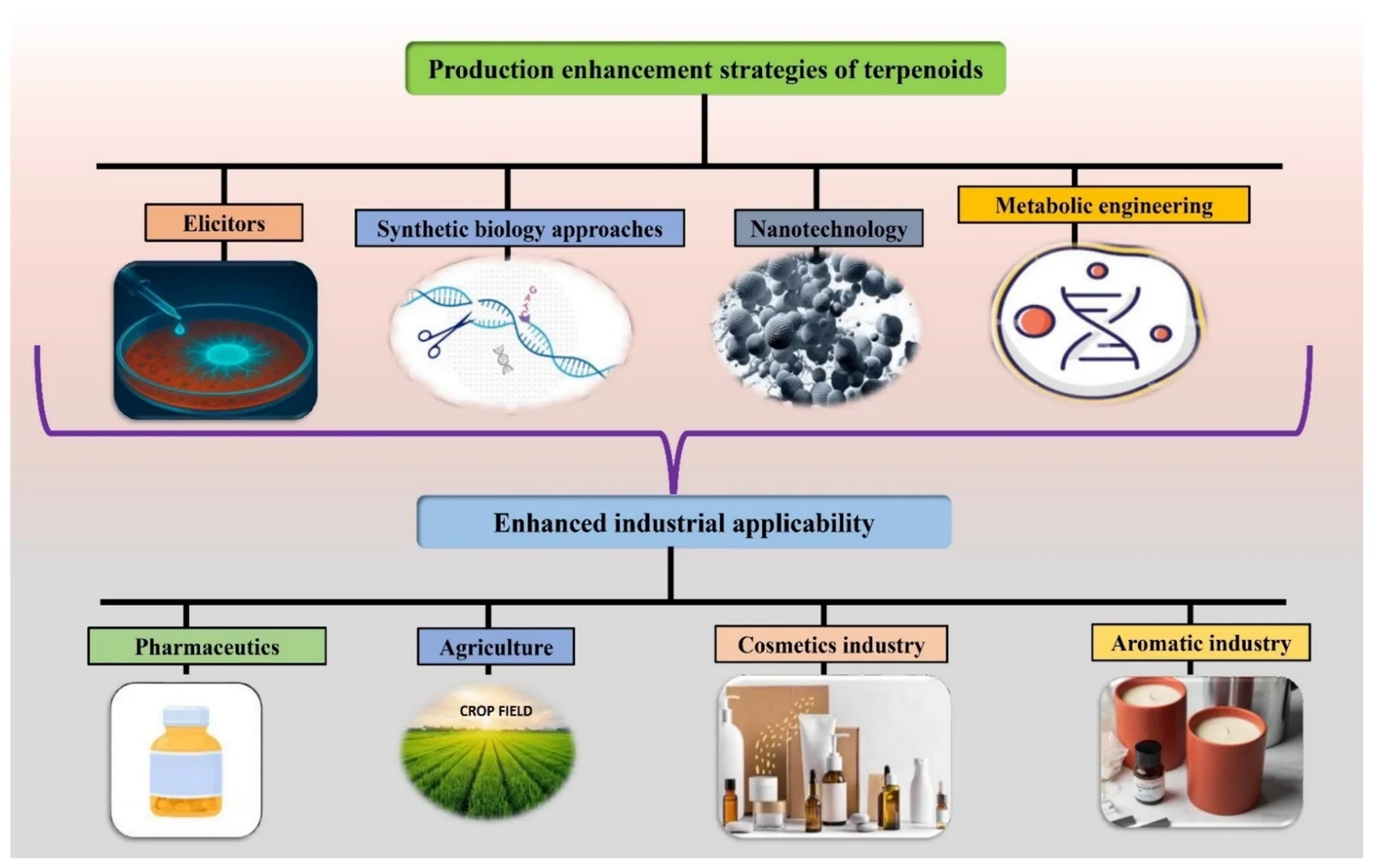
Schematic diagram showing scaling up of production of terpenoids for better industrial applications.

Bioavailability is a crucial determinant of the therapeutic effectiveness of a compound, as it reflects the extent and rate at which an active compound reaches the systemic circulation and becomes available for distribution to its site of action (Figure 7). Although many bioactive compounds possess considerable pharmacological potential, their clinical utility is often limited by poor absorption, rapid metabolism, and restricted distribution within the body [189]. To overcome these limitations, advanced drug delivery systems, including nanotechnology- and polymer-based carriers, have been investigated for their ability to improve the solubility, stability, and targeted delivery of bioactive compounds. In addition to enhancing absorption, some of these systems can provide controlled or sustained release, potentially improving therapeutic efficacy [190].

Many terpenoids exhibit poor aqueous solubility and unfavorable pharmacokinetic properties, particularly because of their hydrophobic or lipophilic nature, which can limit their therapeutic application. Therefore, improving their pharmacokinetic properties through advanced formulation approaches represents an important strategy for facilitating their translation into therapeutic applications. Liposomal encapsulation has emerged as a promising approach for improving the solubility, stability, and delivery of hydrophobic compounds. Paclitaxel, a diterpenoid-derived anticancer agent with very low aqueous solubility, is an important example of a compound for which lipid-based delivery strategies have been extensively investigated [191]. Liposomal formulations prepared using phosphatidylcholine, phosphatidylglycerol, and cholesterol through the thin-film hydration technique have been reported to improve the physicochemical stability and solubility of paclitaxel [192]. Similarly, thymol and menthol have been successfully encapsulated using soybean lecithin-based systems, resulting in improved formulation characteristics and stability [193]. To address the poor aqueous solubility of lupeol, liposomal systems containing phospholipid-based components have also been developed using thin-film dispersion approaches to facilitate its delivery [194]. Collectively, these studies highlight the potential of liposomal formulations to improve the physicochemical and drug delivery properties of hydrophobic terpenoids.

In addition to liposomes, other nanotechnology-based systems can improve solubility and protect bioactive compounds against premature degradation, potentially enhancing their pharmacological activity. Phytosome-based formulations have been developed using phospholipids to improve the delivery of *Ginkgo biloba* constituents, including ginkgolides, with studies reporting enhanced biological effects, such as hepatoprotective and antioxidant activities [195]. Nanoemulsion-based approaches have also been explored for poorly soluble compounds such as andrographolide. Formulations prepared using appropriate surfactants and other excipients can improve compound dispersion and systemic availability and may enhance associated pharmacological effects, including anti-inflammatory activity.

Polymeric nanoparticles represent another promising delivery platform. Nanoparticles developed using PELGE copolymers [monomethyl poly(ethylene glycol)-polylactide-co-glycolide-monomethyl poly(ethylene glycol)] have been investigated for the delivery of ginkgolides A, B, and C and bilobalide using co-precipitation approaches. Incorporation of these compounds into polymeric carriers has been associated with improved pharmacokinetic parameters, including an increased area under the concentration–time curve (AUC), indicating enhanced systemic exposure [196]. Such findings suggest that polymeric delivery systems may help overcome some of the pharmacokinetic limitations associated with poorly soluble natural products.

Metal and metal oxide nanoparticles, including gold, silver, and zinc oxide nanoparticles, have also attracted considerable attention as potential platforms for the delivery or functionalization of natural bioactive compounds. Their physicochemical properties can be tailored for biomedical applications; however, their biocompatibility and toxicity depend strongly on factors such as particle size, surface chemistry, dose, composition, and route of administration. For example, zinc oxide (ZnO) nanoparticles have been synthesized through co-precipitation or green-synthesis approaches using *Andrographis paniculata* extracts containing andrographolide and have subsequently been investigated for their biological activities [197]. In another study, silver nanoparticles (AgNPs) were synthesized using metabolites derived from *Xylaria primorskensis*, and their association with bioactive compounds was investigated as a strategy for improving physicochemical and biological properties [198]. These studies indicate that metal-based nanomaterials may provide useful platforms for the delivery and functional enhancement of natural compounds, although their translational potential requires careful evaluation.

Despite these advantages, metal-based nanoparticles are not without limitations. Their biodistribution, potential accumulation in specific tissues or organs, clearance kinetics, long-term toxicity, and interactions with biological systems require comprehensive investigation before widespread clinical application [199,200]. Similar considerations apply to other nanocarrier systems, for which improvements in solubility or systemic exposure do not necessarily translate directly into improved clinical efficacy.

Overall, advanced formulation strategies, including liposomes, phytosomes, nanoemulsions, polymeric nanoparticles, and metal-based nanomaterials, offer promising approaches for overcoming the poor solubility, stability, and bioavailability of many terpenoids. However, successful clinical translation will require not only improved physicochemical and pharmacokinetic properties but also rigorous assessment of formulation stability, biodistribution, toxicity, scalability, reproducibility, and therapeutic efficacy in well-designed preclinical and clinical studies.

Carrier-based approaches, such as cyclodextrin complexation and solid dispersions, are effective methods for improving the solubility and bioavailability of poorly water-soluble terpenoids. Cyclodextrins, which are cyclic oligosaccharides, form inclusion complexes with hydrophobic molecules, thereby enhancing their aqueous solubility and stability [201]. For example, the andrographolide complex with β-Cyclodextrin (β-CD) exhibited significantly improved solubility and bioavailability [202]. Similarly, thymol synthesized through the sealed heating method in a 1:1 drug-to-carrier ratio demonstrated a higher dissolution rate [203]. Inclusion of linalool with β-Cyclodextrin through co-precipitation not only enhanced solubility but also improved antibacterial activity [204]. Likewise, α-terpineol showed enhanced water solubility and concentration when it was submitted to the co-precipitation technique with (1:1) β-Cyclodextrin [205]. The kneading method has also proven effective, as demonstrated by a 1:1 complex of thymol and carvacrol, which exhibited improved antioxidant properties [206]. Notably, β-carotene complexed with β-Cyclodextrin in a 1:2 molar ratio through kneading exhibited an 18-fold increase in the solubility and dissolution rate [207]. These findings emphasize the utility of cyclodextrin inclusion complexes in optimizing the pharmacokinetic and therapeutic properties of terpenoids for pharmaceutical applications. In parallel, solid dispersion techniques involving hydrophilic carriers and hydrophobic drug molecules have also been employed to overcome solubility limitations. This strategy has demonstrated efficacy in increasing the dissolution rates and bioavailabilities of multiple bioactive compounds, although stability may remain a concern. For instance, andrographolide combined with PVP-K30 and kolliphor through solvent evaporation showed a 3-fold increase in bioavailability [208]. Ginkgolide B, when dispersed with PVP-K30, urea, and HPMC, showed a notable enhancement in water solubility [209]. Similarly, glycyrrhetinic acid exhibited improved solubility and an improved dissolution rate after being processed with Kollidon^®^ VA64 using the hot-melt extrusion technique. These results demonstrate how the solid dispersion approach can be used to improve medication formulations for increased therapeutic efficacy [210].

Co-crystals have emerged as a promising strategy for enhancing the solubility and dissolution characteristics of poorly soluble terpenoids by forming stable supramolecular complexes with suitable conformers. This approach involves the non-covalent interaction between an active pharmaceutical ingredient (API) and a conformer, leading to improved physicochemical properties without altering the molecular structure of the drug. For instance, diosgenin complexed with piperazine in a 2:1 molar ratio demonstrated a 1.5-fold increase in solubility, indicating the potential of this method for steroidal terpenoids [211]. Similarly, a 1:1 co-crystal of artemisinin with amodiaquine dihydrochloride dihydrate resulted in a 33.41% enhancement in its solubility [212]. Other hydrophobic compounds have also shown marked improvement in both solubility and bioavailability when co-crystallized with pyrogallol and resorcinol in a 1:1 ratio [213]. Furthermore, the combination of andrographolide with salicylic acid (1:1) led to a 12-fold increase in solubility and a 3-fold enhancement in the dissolution rate [214]. These results highlight how co-former-assisted formulation techniques can improve the delivery of terpenoid-based drugs.

Glycosylation, the enzymatic attachment of sugar moieties to terpenoid structures, has proven to be an effective strategy for improving the water solubility, bioavailability, and overall pharmacological performance of these compounds. This biochemical modification not only improves hydrophilicity but also alters pharmacokinetic properties and enhances interaction with cellular membranes. For example, glycosylation of andrographolide using β-Galactosidase results in the formatting of 19-O-β-D-galactosyl-andrographolide, which demonstrates significantly enhanced solubility and improved antibacterial activity compared to the parent compound [9]. Similarly, celastrol, known for its potent but poorly soluble and toxic profile, was converted into Celastrol-29-O-β-glucoside using Bacillus GT (BsGT110). This modification yielded a 53-fold increase in aqueous solubility, along with markedly improved bioavailability and antioxidant activity [215]. In another instance, glycosylation of ganoderic acid A by *Bacillus* sp. GA A07 led to the formation of ganoderic acid A-15-O-β-D-glucoside. This derivative exhibited superior anticancer and anti-inflammatory properties due to enhanced solubility and improved systemic availability. Collectively, these glycosylation-based strategies effectively address the inherent limitations of hydrophobicity in terpenoids, offering a viable approach to develop more potent and clinically applicable therapeutics. Hence, these strategies are efficient at enhancing the bioavailability of terpenoids in test models, thereby overpowering their drawbacks, which creates a research gap in translational research.

## 10. Conclusions and Future Prospects

Terpenoids represent a wide range of natural compounds isolated from plants, microorganisms and marine organisms, which play an essential role in physiological processes and are used in various pharmaceutical and industrial applications. The activities of terpenoids in fields such as infectious diseases, metabolic conditions, cancer, agriculture, foods and cosmetics show the broad application range of the compounds. Nevertheless, the practical application of many terpenoids suffers due to the low content in natural sources, complex structure, instability, insolubility, low bioavailability and problems related to consistent large-scale production. The development in the areas of extraction, elicitation, metabolic engineering, synthetic biology and formulations opens up new possibilities for improving the production and performance of terpenoids.

The further development of terpenoid products will include increasing the production efficiency, quality control, safety evaluation, bioavailability and clinical evidence. The solutions to these aspects will be required for the successful transformation of promising terpenoids to reliable pharmaceuticals and industrial products.

## Figures and Tables

**Table 2:** Terpenoids from various sources which have undergone clinical trials with their efficacies as particular pharmaceutical compounds.

Terpenoid Compounds	Source	Chemical Structure	Target Conditions	Trial Phase	Findings	Ref.
Artemisinin (C_15_H_22_O_5_)	*Artemisia annua*	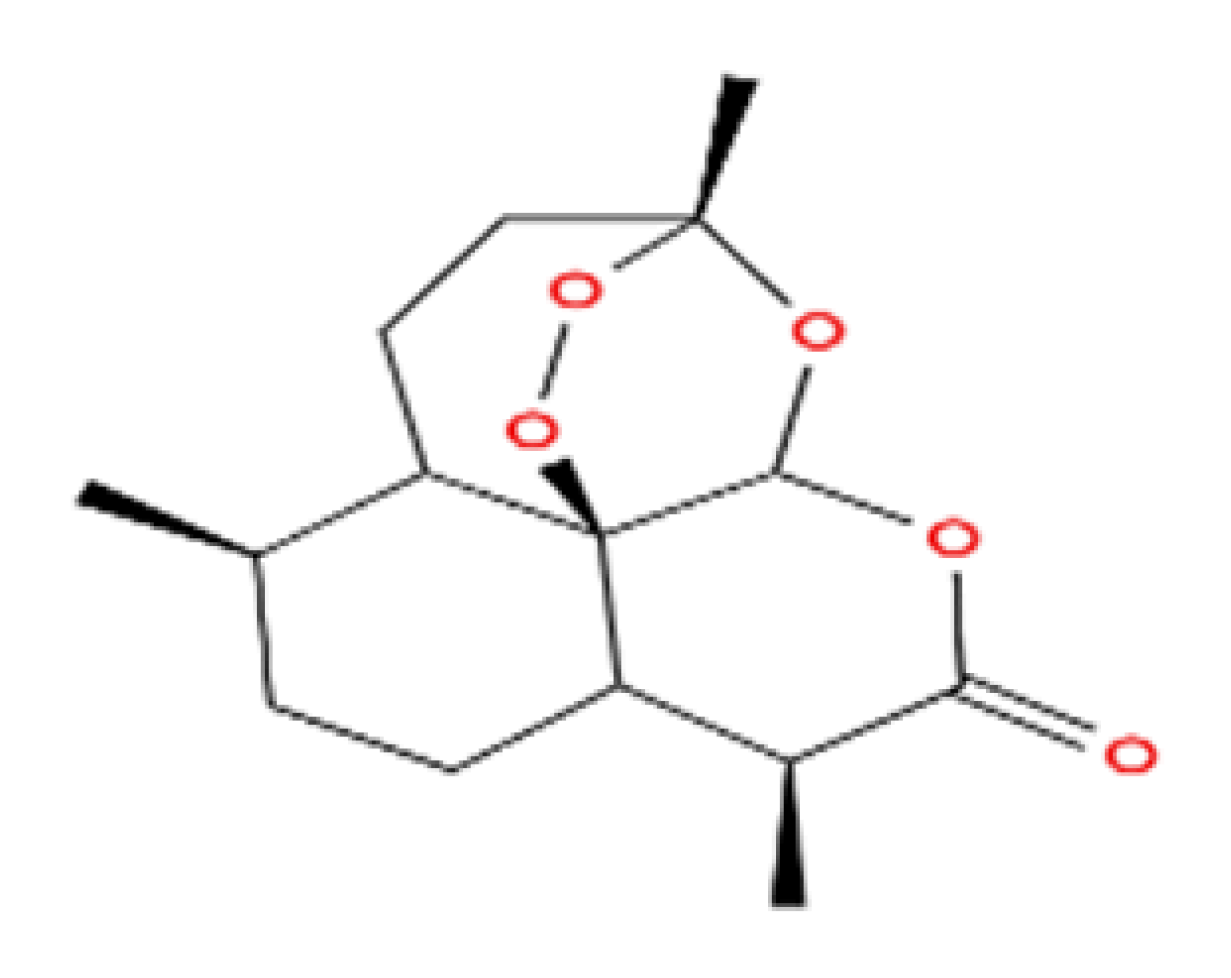	Malaria, cancer	Phase II/III	Effective antimalarial; under cancer evaluation	[97]
Boswellic acids (C_30_H_48_O_3_)	*Boswellia serrata*	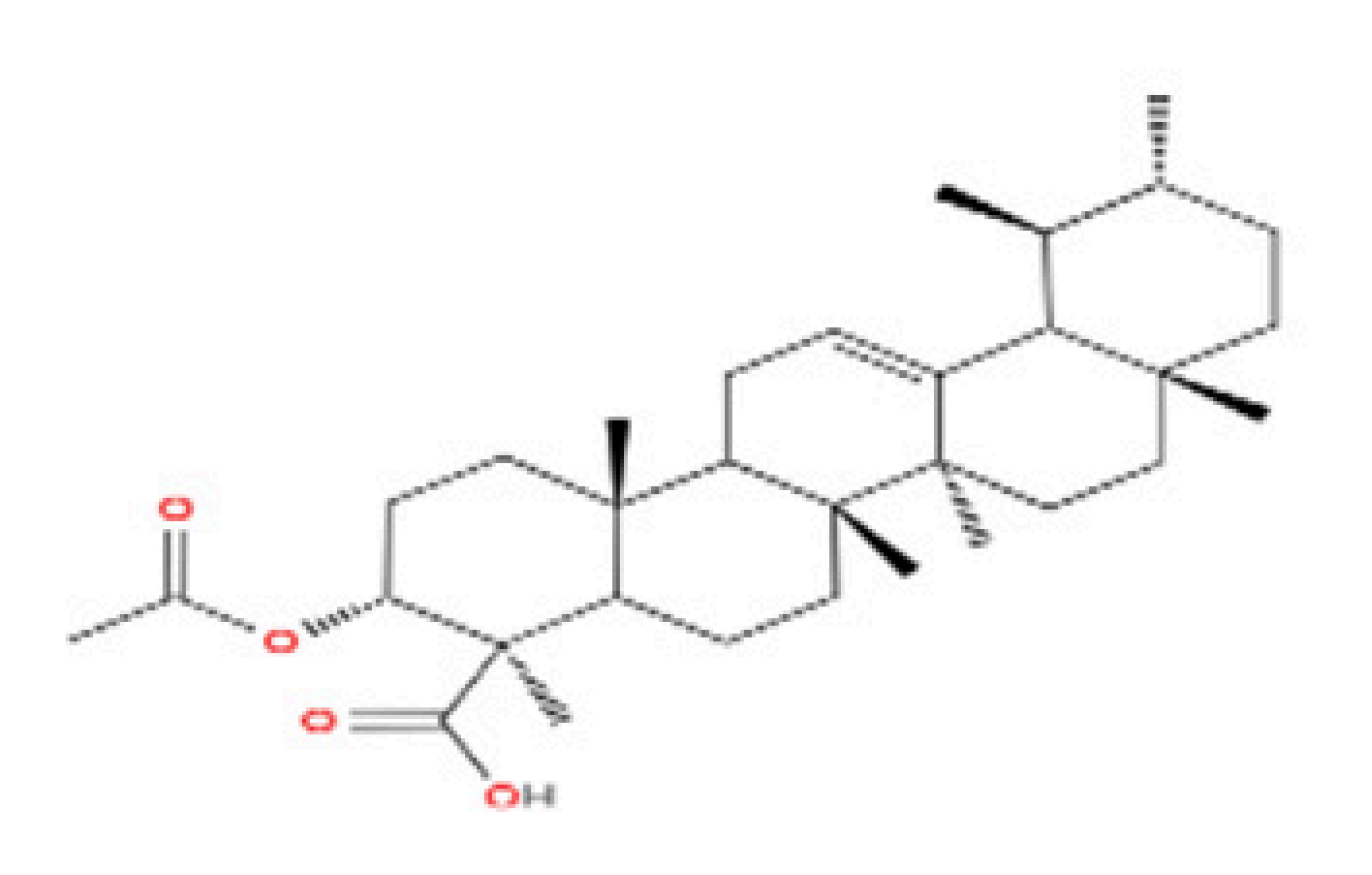	Osteoarthritis, IBD	Phase II	Reduced joint pain and inflammation	[97]
D-limonene (C_10_H_16_)	Citrus peel oil	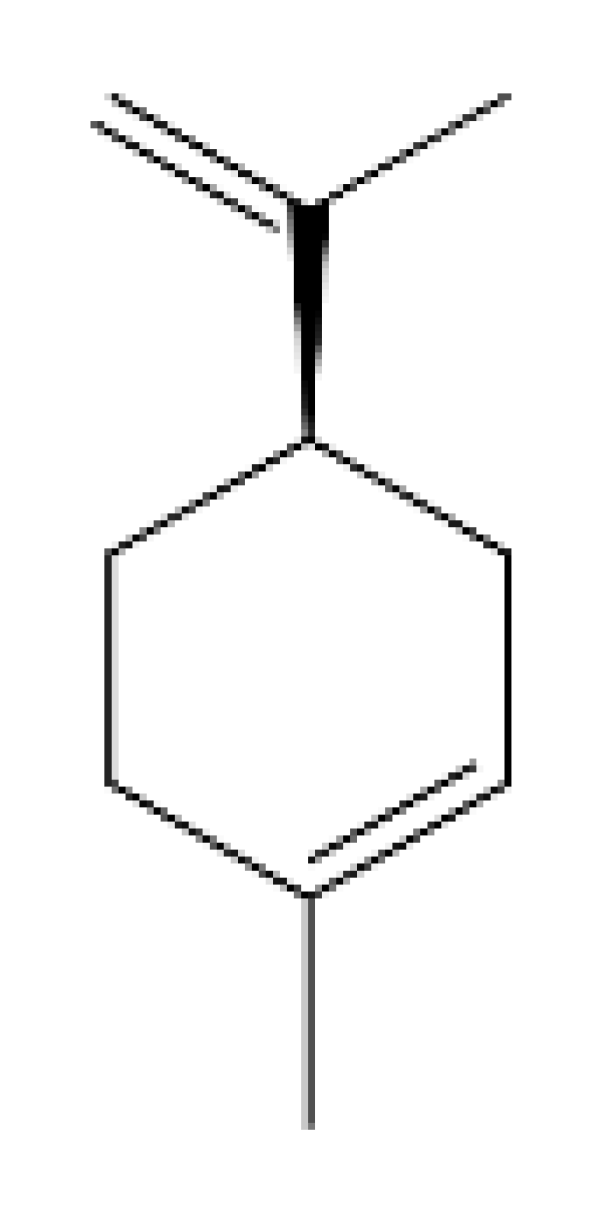	GERD, breast cancer	Phase II	Safe; demonstrated chemopreventive activity	[97]
Ginkgolides (C_20_H_24_O_9_)	*Ginkgo biloba*	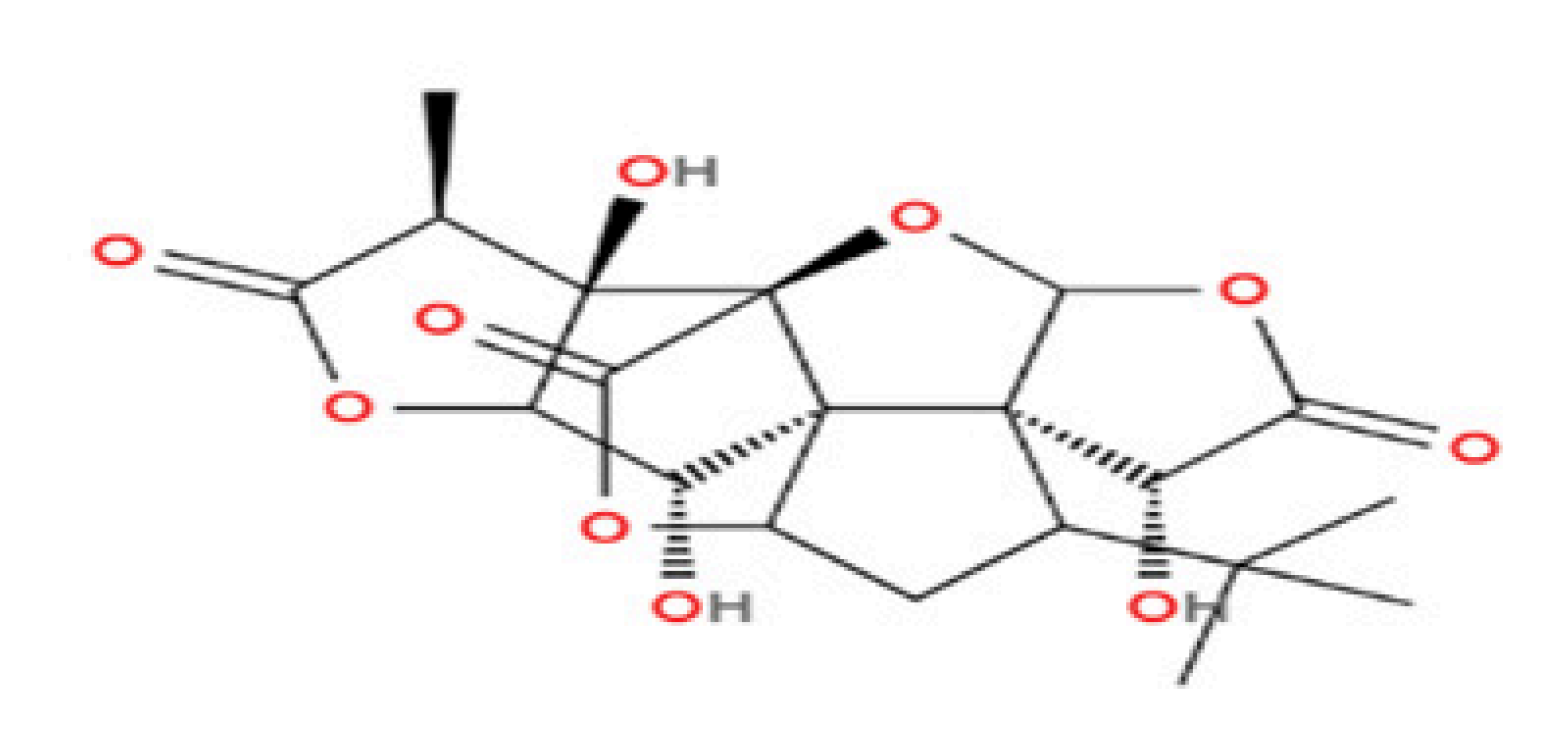	Cognitive decline, dementia	Phase III	Improved memory; no adverse neurological effects	[97]
Lupeol (C_30_H_50_O)	*Ricinus communis*, *Acalypha indica*	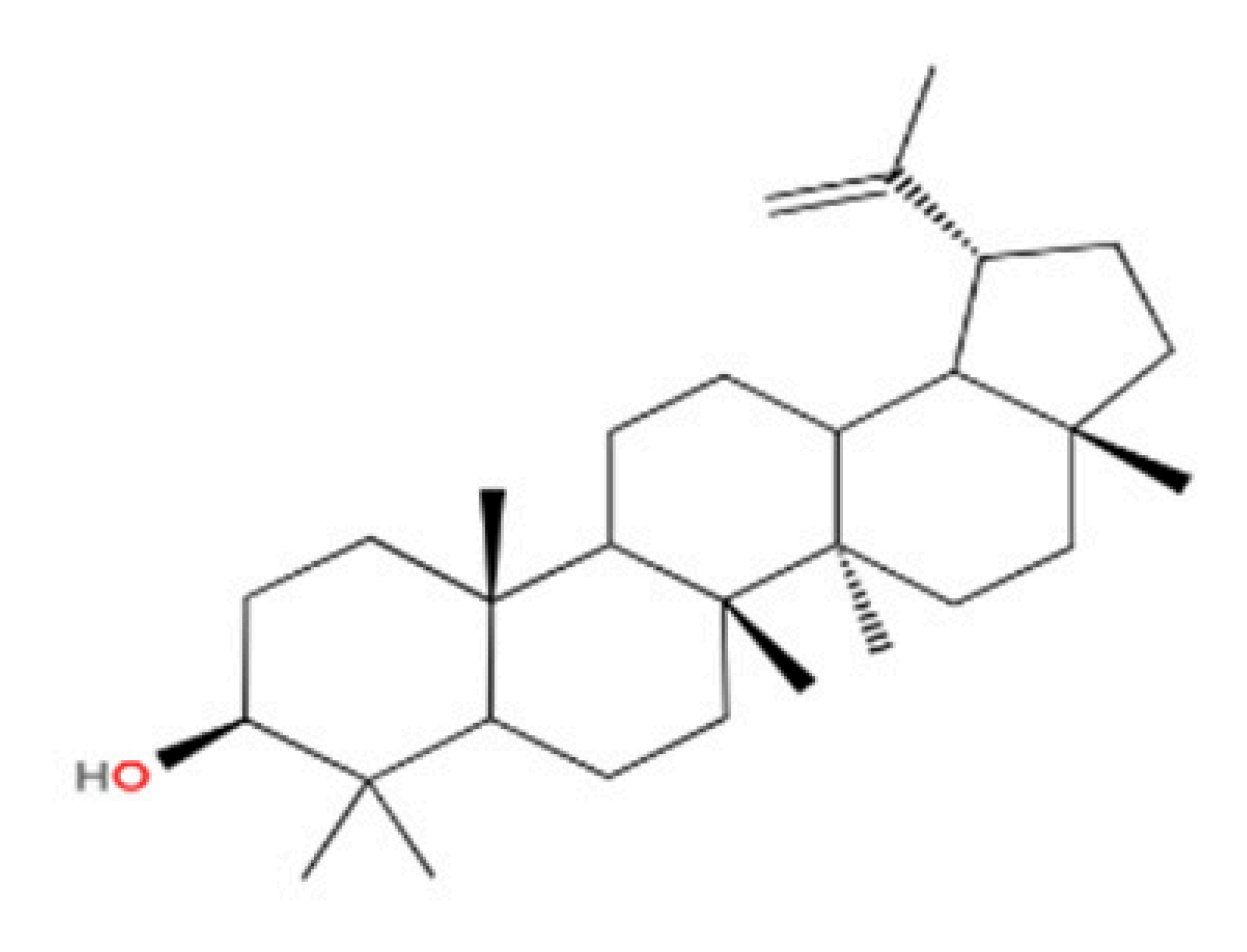	Prostate and skin cancer	Phase I	Induced apoptosis; low systemic toxicity	[97]
Betulinic acid C_30_H_48_O_3_	*Gratiola officinalis* L.	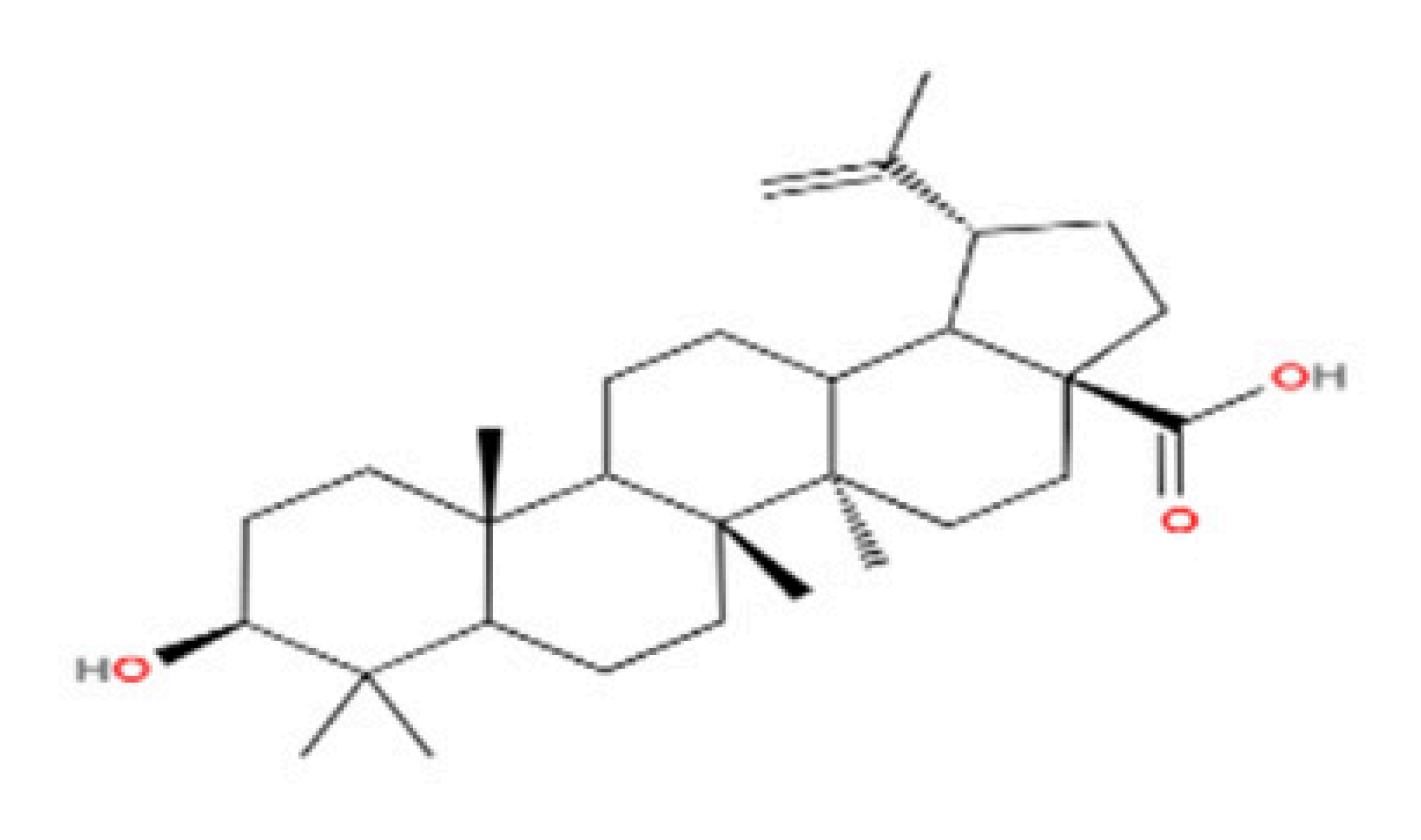	Cancer	In vitro and in vivo	Anticancerous; induced apoptosis and inhibited metastasis	[98]
Andrographolide C_20_H_30_O_5_	*Andrographis paniculata* leaves	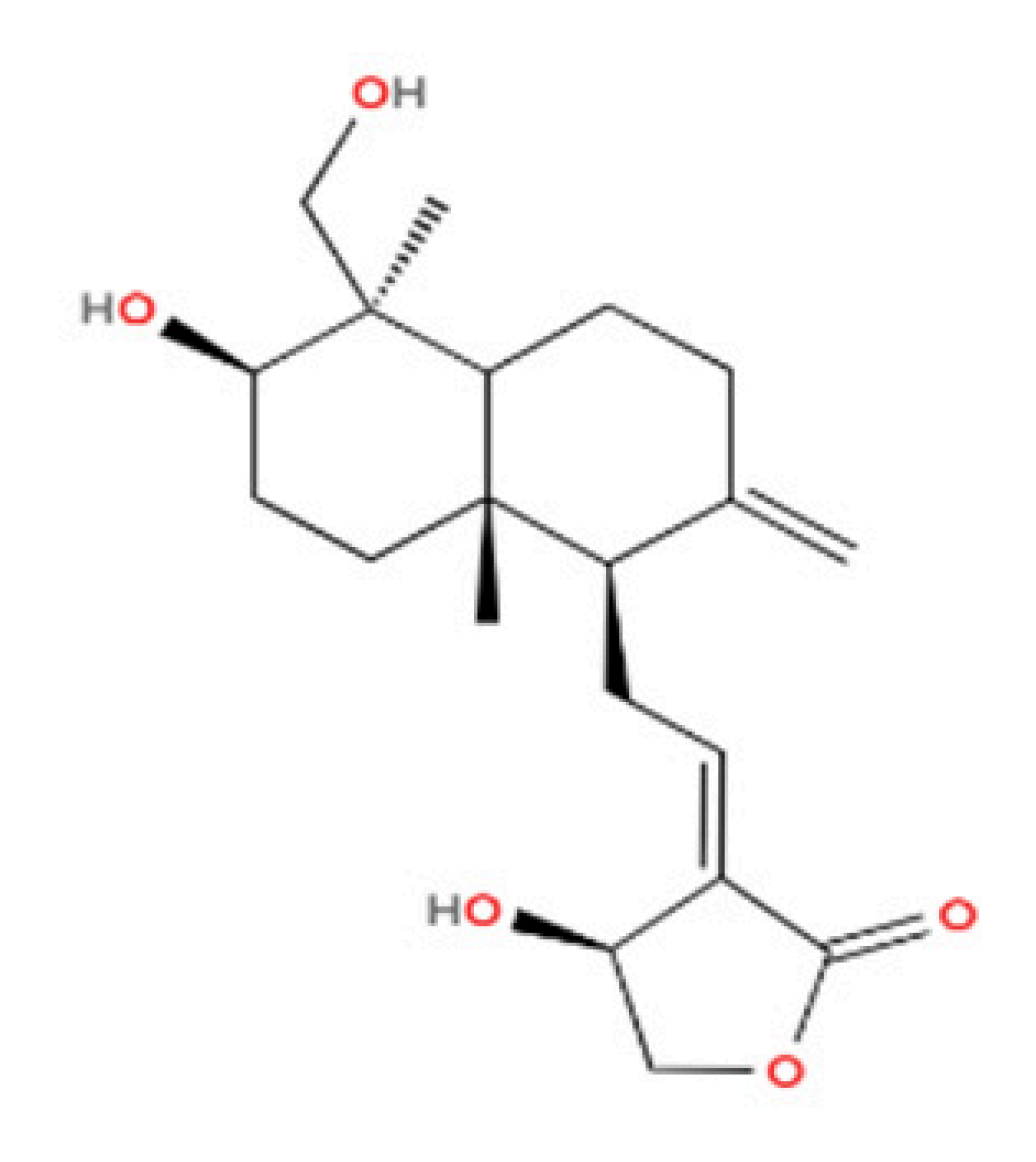	Diabetes	Diabetic rats	Lower level of glucose in plasma, glucose utilization enhanced, inhibits retinal angiogenesis, and normalizes estrous cycles	[99]
Resin (olibanum gum) mixture	*Boswellia* species		Diabetes	T2DM patients; unspecified dose and duration	Reduced glycosylated hemoglobin and fasting plasma glucose	[100]

**Table 3:** Common terpenoids as commercial medicines and their medicinal activity.

Terpenoid	Source	Commercial Product Name	Application Area	Remarks	Ref.
Paclitaxel	*Taxus brevifolia*	Taxol^®^, Abraxane^®^ (Bristol-Myers Squibb Company, Princeton, NJ, USA)	Oncology	Used for breast, lung and ovarian cancers	[91]
Artemisinin	*Artemisia annua*	Coartem^®^, artesunate (Novartis AG, Basel, Switzerland; artesunate API: Sigma-Aldrich, St. Louis, MO, USA)	Antimalarial	WHO-listed essential antimalarial drug	[119]
Boswellic Acids	*Boswellia serrata*	Sallaki^®^, shallaki^®^, 5-loxin^®^ (Sundyota Numandis Group, Ahmedabad, Gujarat, India)	Anti-inflammatory	Osteoarthritis and joint care	[120]
Limonene	Citrus peel oil	Natural cleaners, citrus supplements	Food, fragrance, anticancer	Also used in ecofriendly solvents	[121]
Menthol	*Mentha arvensis*	Vicks^®^, IcyHot^®^, Bengay^®^ (Kenvue Brands LLC, Summit, NJ, USA)	Analgesic, cooling agent	Topical ointments and throat lozenges	[122]
Thymol	*Thymus vulgaris*	Listerine^®^, Eucalyptol^®^ blends (Kenvue Brands LLC, Summit, NJ, USA; Eucalyptol: Sigma-Aldrich, St. Louis, MO, USA)	Oral hygiene	Antiseptic and flavoring agent	[123]
Lupeol	*Ricinus communis*, mango bark	Herbal ointments, cosmeceuticals	Anti-inflammatory, skin care,	Anti-acne and anti-aging formulations	[124]
Carvacrol	*Origanum vulgare*	FoodSafe^®^, Greenseal^®^ (natural preservative) (Kalsec Inc., Kalamazoo, MI, USA)	Food safety	Antibacterial against food-borne pathogens	[125]
Pinene	-	Eucalyptus pinene enteric soft capsules, nateglinide (antidiabetic drugs) (Grand Pharmaceutical Group, Wuhan, Hubei, China; nateglinide: Novartis Pharmaceuticals Corporation, East Hanover, NJ, USA)	Antitumor, antifungal, and anti-allergic (reduces the symptoms of allergic rhinitis); improves ulcers	Pharmacologically active and helps intestinal peristalsis in rats	[125]
β-Elemene	-	Elemene oral milk, elemene injection (Dalian Huali Jingang Pharmaceutical Co., Ltd., Dalian, Liaoning, China)	Lessens mitosis (growth) of tumors and cells and induces their apoptosis	Suppresses microbial intestinal–brain axis imbalance	[126]
D-Limonene	-	Qiulin limonene capsules, eucalyptus pinene enteric soft capsules (Grand Pharmaceutical Group, Wuhan, Hubei, China)	Antitussive, expectorant, antibacterial effect	Restricts action of food spoilage fungi and food-borne pathogens, regulating intestinal tract	[93]
Geraniol	-	Geraniol soft capsules, geraniol (Sigma-Aldrich, St. Louis, MO, USA)	Manages ulcers, neurasthenia, skin aging, thrombosis, arterial sclerosis and immune deficiency	Antitoxic compound, restrains *Salmonella typhimurium* infection; antibacterial and anti-inflammatory, sustains intestinal microecological balance and helps intestinal health	[127]
β-Caryophyllene	-	β-caryophyllene API (Sigma-Aldrich, St. Louis, MO, USA)	Local anesthesia, treatment of colitis	Antioxidant, antibacterial and anti-inflammatory activities	[128]

**Table 4:** Reported toxicity and safety concerns associated with selected terpenoids.

Terpenoid	Toxic Effect	Tested Organism	Exposure	Ref.
α-terpinene	Embryotoxicity, neurotoxicity, hepatic oxidative stress, genotoxicity	Rats, mice; cultured cells	Dose-dependent experimental exposure	[143]
Citral	Genotoxicity, DNA damage, embryotoxicity	Rats; human-derived cells	Concentration-dependent in vitro and experimental exposure	[143]
Linalool/limonene	Irritation and sensitization, particularly with concentrated topical exposure	Humans/other experimental models	Concentration- and exposure-dependent	[143]
Menthol	Gastrointestinal and CNS toxicity, including dizziness, convulsions and coma at excessive exposure	Experimental models; human/clinical reports	High-dose exposure	[148]
Pulegone	Hepatotoxicity; reproductive and tissue toxicity	Mice; humans	High-dose experimental exposure; human cases reported	[143]
Thujone	Neurotoxicity, behavioral changes, hepatic and renal effects	Mice, rats; human liver preparations	Dose-dependent experimental exposure	[143]
Tea tree oil terpenes	Skin irritation, allergic/adverse reactions; poisoning following ingestion	Humans	Adverse-event reports; exposure amount variable	[149]
Camphor	Neurotoxicity; hepatotoxicity	Humans; experimental models	Toxicity associated with high exposure/ingestion	[143]

**Table 5:** Comparative analysis of strategies to enhance terpenoid production.

Strategy	Mechanism	Advantage	Limitations	Reference
Elicitor application	Stimulates secondary metabolism via biotic/abiotic stress	Cost-effective, non-transgenic	Results can be inconsistent; needs optimization	[154]
Genetic engineering (CRISPER, overexpression)	Alters metabolic pathway to upregulate key biosynthetic genes	High specificity, permanent changes	Regulatory hurdles; off-target risks	[155]
Cell/organ culture	In vitro systems for scalable production	Controlled conditions, year-round yield	Expensive setup; limited scalability in some systems	[156]
Nanoparticle-mediated elicitation	Nanoparticles act as abiotic stressors to trigger metabolic production	Highly efficient at low doses	Toxicity concern; less understood mechanism	[157]
Organelle engineering	Directs enzymes or precursors to organelles	Improves local concentration; spatial control	Technically complex; required robust host systems	[155]
Synthetic biology (pathway modularization)	Designs and assembles custom pathways in microbial chassis	Precision control, multi-product potential	Requires sophisticated tools; limited by host toxicity	[158]

**Table 6:** Enhanced production of terpenoids using organelle compartmentalization strategies.

Products	Organelle	Organism	Major Engineering Strategies	Ref.
β-amyrin	ER	*S. cerevisiae*	Knockout PAH1	[161]
	LDs	*S. cerevisiae*	Semi-rational modification of MdOSC1, enhancement of MVA pathway and upregulation of DGA1 expression	[162]
Squalene	ER	*S. cerevisiae*	Overexpression of INO2	[163]
	LDs	*Y. Lipolytica*, *S. cerevisiae*	Overexpression of tHMG1 and DGA1	[164]
	Peroxisome	*S. cerevisiae*	Hybridization of strain engineered for cytoplasmic and peroxisomal modifications	[165]
	Plasma membrane	*E. coli*	Overexpression of TSR, which expands membrane volume	[166]
Lycopene	LDs	*Y. Lipolytica*	Inhibition of β-oxidation pathway and modification of terpenoid synthesis pathway	[167]
		*S. cerevisiae*	Enhancement of TAG biosynthetic pathway with adjustment of its fatty acyl profile	[168]
	Peroxisome	*P. pastoris*	Direct heterologous carotenoid producing enzymes to peroxisomal compartments	[169]
Ginsenoside	ER	*S. cerevisiae*	Overexpression of INO2	[164]
	LDs	*S. cerevisiae*	Localization of protopanaxadiol synthase to lipid droplets (LDs) and enhancement of TAG pathway	[170]
Patchoulol	Mitochondrion	*S. cerevisiae*	Incorporation of DMAPP biosynthesis pathway into mitochondria compartments	[171]
Amorphadiene	Mitochondrion	*S. cerevisiae*	Simultaneous overexpression of tHMG1, mitochondria-targeted heterologous FDP synthase and amorphadiene synthase	[172]

## Data Availability

No new data were created or analyzed in this study. Data sharing is not applicable to this article.
